# Psychosocial support interventions to improve treatment outcomes for people living with tuberculosis: a mixed methods systematic review and meta-analysis

**DOI:** 10.1016/j.eclinm.2023.102057

**Published:** 2023-06-27

**Authors:** Claire Maynard, Shema Tariq, Giovanni Sotgiu, Giovanni Battista Migliori, Martin van den Boom, Nigel Field

**Affiliations:** a UCL Institute for Global Health, UK; b Clinical Epidemiology and Medical Statistics Unit, University of Sassari, Italy; c Istituti Clinici Scientifici Maugeri IRCCS, Tradate, Italy; d WHO Regional Office for the Eastern Mediterranean Region, Cairo, Egypt; e Centre for Molecular Epidemiology and Translational Research, UCL Institute for Global Health, UK

**Keywords:** Tuberculosis, Psychosocial, Complex intervention

## Abstract

**Background:**

People with tuberculosis (TB) face multi-dimensional barriers when accessing and engaging with care. There is evidence that providing psychosocial support within people-centered models of care can improve TB outcomes, however, there is limited consensus on what works. It remains important for such interventions to be rigorously assessed, and mixed methods systematic reviews are one way of synthesising data for policy makers to be able to access such evidence. Mixed methods reviews take a complexity perspective, with qualitative data being used to contextualise the quantitative findings and giving an insight into how interventions are contingent on variations in design and context.

**Methods:**

Five electronic databases were searched from January 1 2015 to 14 January 2023 for randomised controlled trials, quasi-experimental trials, cohort studies and qualitative studies of interventions providing psychosocial support (material and/or psychological-based support) to adults with any clinical form of active TB. Studies with inpatient treatment as the standard of care were excluded. Quantitative studies reporting pre-specified standard TB outcomes were eligible. In line with established mixed methods review methodology, a convergent parallel-results synthesis design was followed: quantitative and qualitative syntheses were distinct and carried out using appropriate methods. A convergent coding matrix was then used to integrate the results. The protocol was registered on PROSPERO (CRD42021235211).

**Findings:**

Twenty-three studies of interventions were included (12 quantitative, 10 qualitative, and 1 mixed methods study) were included. Most studies were conducted in low-and middle-income countries with a high-burden of TB. Three explanatory and contextual middle-range theories from the integration of qualitative and quantitative data were developed: effective interventions provide multi-dimensional support; psychological-based support is transformative but there is insufficient evidence that it improves treatment outcomes on its own; intervention delivery shapes a logic of care.

**Interpretation:**

This review takes a complexity perspective to provide actionable and timely insight to inform the design and implementation of locally-appropriate and people-centered psychosocial support interventions within national TB programmes.

**Funding:**

There was no funding source for this study.

**Research in context**

**Evidence before this study**

We searched PubMed in January 2020 for English language systematic reviews focusing on psychosocial interventions for people with TB and supplemented the search with any systematic reviews known to the authors. The limited number of systematic reviews identified focused on the quantitative impact on standard TB outcomes of single components of psychosocial support interventions, primarily from non-randomised intervention studies. It emerged that while there are multitudes of studies of complex psychosocial support interventions, variable in both their content and the systems in which they operate, conventional approaches to evidence generation have thus far limited the ability make actionable recommendations to support policy change.

**Added value of this study**

In response, we conducted a mixed methods systematic review and meta-analysis using a convergent parallel-results synthesis design to integrate quantitative and qualitative data. Mixed methods reviews take a complexity perspective, with qualitative data being used to contextualise the quantitative findings and giving an insight into how interventions are contingent on variations in design and context. We developed three actionable middle-range theories which articulate which components of psychosocial support interventions work, their mechanisms of action, and the conditions that need to be satisfied for successful implementation.

**Implications of all the available evidence**

Given the detrimental and compound impact of the COVID-19 pandemic on people living with TB and health systems’ resources, this review provides actionable and timely insight to inform the design and implementation of locally-appropriate and people-centered psychosocial support interventions within national TB programmes.

## Introduction

Tuberculosis (TB) is the second highest cause of death from an infectious disease after COVID-19 globally, with the majority of cases in low- and middle-income settings.^1^ Despite being preventable and curable, the intersection of complex biopsychosocial determinants of TB (e.g., poverty, undernutrition, HIV infection, smoking and diabetes), coupled with long and potentially toxic drug regimens, necessitates a multi-sectoral approach to care. While TB medication is widely provided by universal health coverage, people with TB face barriers to care including travel costs, loss of income as a result of side effects and sickness absence, TB-related stigma and mental health issues.^2^ Without sufficient support, these barriers can limit a person's access to and engagement with care for the duration of their treatment. This increases risk of community transmission, acquisition of drug-resistant TB (DR-TB) and death.^3^

The provision of psychosocial support (material support and/or psychological-based support) within a people-centered model of care was proposed by the World Health Organization's (WHO) End TB Strategy as a key strategy for ending the TB epidemic within the period 2015–2035.^4^ However, constrained resources and shifting priorities of health systems have hindered widespread uptake and successful implementation of such models within National TB Programmes (NTPs). A recent scoping review reported a paucity of person-centered approaches to TB care in low- and middle-income and BRICS countries (Brazil, Russia, India, China and South Africa).^5^ The COVID-19 pandemic compounded these challenges, causing a sharp reduction in the number of people with TB seeking care, a drop in diagnoses and diagnostic capacity and less care being available if sought.^6^^,^^7^ Pandemic-related disruption is estimated to have resulted in an additional 100,000 global TB deaths in 2020 compared to 2019.^1^ Continued disruption to services and increased biopsychosocial vulnerabilities of people living in high-burden settings has reversed progress and pushed global TB targets off track.

To mitigate the impact of the COVID-19 pandemic, it is essential that sufficient resources are channelled to NTPs and that people-centered models of care are prioritised. However, there is a paucity of evidence on what works in terms of psychosocial support and how to design and implement effective and appropriate programmes. In this systematic review we aim to understand active components of effective psychosocial support interventions for people living with TB, their mechanisms of action, and the conditions to be satisfied for successful implementation.

## Methods

This systematic literature review was reported in accordance with the Preferred Reporting Items for Systematic Reviews and Meta-Analyses guidelines.^8^ The field of mixed methods systematic reviews is emergent, however this approach is recognised as having particular strengths when informing policy. By synthesising literature on both efficacy and important programmatic factors such as feasibility, acceptability and experience, mixed methods reviews are able to provide a robust evidence base from which to make decisions.^9^, ^10^, ^11^

**Fig. 1:**
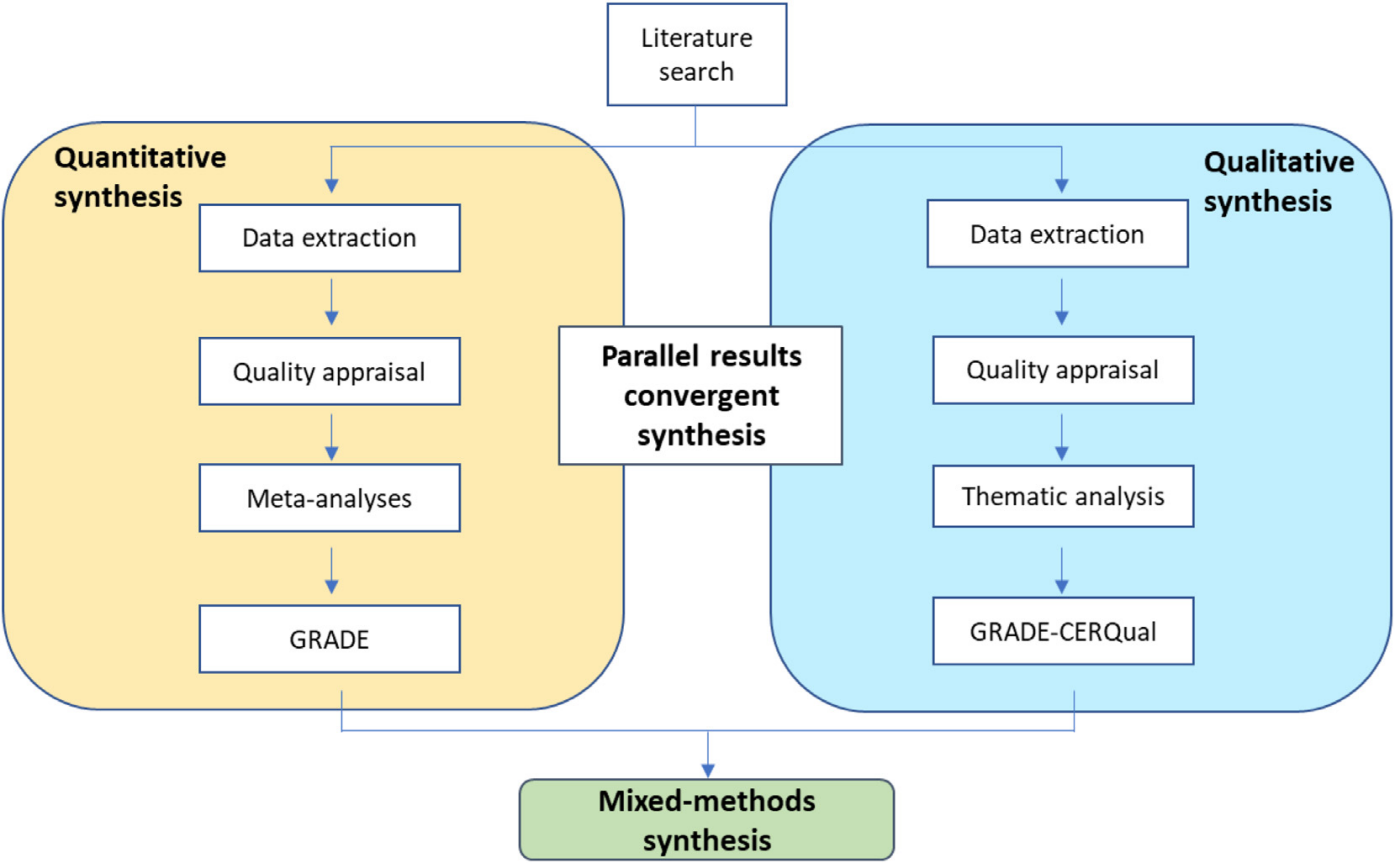
Diagram of the parallel results convergent synthesis design of this review.

The protocol was prospectively registered on PROSPERO (CRD42021235211). We employed a convergent synthesis design; we synthesised findings from quantitative and qualitative literature separately, before integrating these using a matrix to identify where quantitative and qualitative findings supported (convergence), refuted (dissonance), added (complementarity) to each other, or if one synthesis provided insufficient or no evidence (silence)^12^ (Fig. 1).

### Search strategy and selection criteria

Five electronic databases (CINAHL, Cochrane Central Register of Controlled Trials CENTRAL, EMBASE, MEDLINE and PsycINFO) were searched on February 4 2021, and again on 14 January 2023. Peer-reviewed and English language randomised controlled trials, quasi-experimental trials, non-randomised studies of interventions (NRSIs) (including prospective and retrospective cohort studies and before-and-after studies), and qualitative studies of interventions providing psychosocial support for adults (16 years old and above) diagnosed with any clinical form of active TB, including those with HIV co-infection, were eligible. Databases were searched from January 1 2015 to optimise the relevance of the review to standards of care and the policy context following the End TB Strategy (2015–2035). The full eligibility criteria and search strategy are presented in Supplementary Tables S2 and S3.

Psychosocial support was defined as a combination of psychological-based support (for example: counselling sessions, peer-group support and health education) and/or material support (for example, cash-transfers, transportation vouchers, food vouchers, food packages or supplements).^13^ Studies providing either form of support, or both, were eligible for inclusion. To ensure that this review builds on the evidence demonstrating the effectiveness, cost-effectiveness and acceptability^14^, ^15^, ^16^, ^17^, ^18^ of delivering care in outpatient settings, we chose to exclude studies where the standard setting of care was inpatient settings. Interventions providing variations of directly observed therapy (DOT) or interventions aimed at providing medication reminders only, such as treatment ‘tracers’ or digital monitors were not eligible as these have been comprehensively reviewed.^2^^,^^3^^,^^19^ Studies of interventions not directly aimed at people with TB, for example training for healthcare workers, were excluded.

Quantitative studies reporting at least one mutually exclusive standard TB outcome (treatment success, treatment failure, death, loss-to-follow-up (LTFU) (Supplementary Table S1) were included where odds ratios were presented or could be calculated. Study authors were contacted in cases where details were missing from eligible studies or if full texts could not be retrieved. The search was carried out by CM. Ten percent of title and abstracts and all full-texts were screened independently against quantitative and qualitative inclusion criteria by two additional reviewers (ST and GS) and any eligibility uncertainties were resolved through discussion. Quantitative data extraction was carried out by CM and reviewed for accuracy by GS. Qualitative data extraction was carried out by CM and a second reviewer (ST) independently extracted data from 50% of the studies.

### Quality assessment and GRADE

Included articles were assessed for quality using peer-reviewed and piloted tools: Cochrane risk of bias (RoB) tool for randomised controlled trials (Supplementary Table S4); ROBINS-I for non-randomised studies (Supplementary Table S5); and Critical Appraisal Skills Programme (CASP) checklist for qualitative studies (Supplementary Table S6). Quality assessment was carried out by one reviewer (CM) and reviewed by a second reviewer (GS and ST, for qualitative and quantitative, respectively); any discrepancies were resolved through discussion. Studies at critical risk of bias in any domain were excluded at this stage.

The quality of the evidence for quantitative outcomes and qualitative themes was assessed using GRADE (Supplementary Tables S7–S10) and GRADE-CERQual (Supplementary Table S11), respectively. Inconsistency was based on visual inspection of forest plots and the I^2^ statistic, according to Cochrane standards: 50–90% (serious inconsistency); 75–100% (very serious inconsistency).^20^ In line with GRADE methodology, imprecision was examined using sample size and default thresholds of appreciable benefit or appreciable harm at 0.8 and 1.25. Outcomes were downgraded for imprecision: once if sample size <300; once if 95% confidence intervals (CI) crossed the line of no effect and one threshold for appreciable benefit or harm; twice if 95% CI crossed the line of no effect and both thresholds.

### Data analysis

Quantitative and qualitative analyses were undertaken separately and carried out using appropriate methods. For the quantitative synthesis, dichotomous data for TB outcomes of interest were presented as unadjusted odds ratios with 95% CI. There was insufficient information available to report adjusted odds ratios for all NRSIs; an alternative approach of using unadjusted estimates was taken in preference to excluding these studies. Type of support was used to stratify the data and where appropriate, results pooled and random-effects meta-analyses conducted to account for inherent differences in study populations, intervention design and settings within these groups. Heterogeneity was explored by visual inspection of data, the I^2^ statistic, and by study design subgroups. Differences between subgroups was assessed by visual inspection of confidence intervals. Statistical analysis was carried out using Review Manager 5.3 software. Given the limited number of quantitative studies included we did not explore heterogeneity further through pre-specified subgroup analysis and instead examined patterns in the data narratively.

Qualitative data was extracted *verbatim* from the results and discussion sections of qualitative and mixed method studies and analyzed thematically. Two reviewers (CM and ST) independently familiarised themselves with the data, categorised them by type of support and inductively coded findings into descriptive themes. These themes were then further categorized as ‘mechanisms of action’ or ‘intervention barriers’. Three reviewers (CM, ST and NF) discussed the descriptive themes and selected the socio-ecological framework model (SEM) to organise the ‘mechanism of action’ themes at the individual, community and interpersonal and structural levels to reflect the multi-dimensional nature of these interventions.

Findings from the quantitative and qualitative syntheses were integrated using a convergence coding matrix^12^ to contextualise the findings in relation to different types of psychosocial support and detect the level of agreement. We acknowledge problematic western epistemic paradigms and avoid realist constructions of what works, for whom and in what settings, given that we cannot fully appreciate intersectionality within the methodological constraints of this review and our limited lived experience.

### Role of the funding source

There was no funding source for this study.

## Results

### Characteristics of included studies

**Fig. 2:**
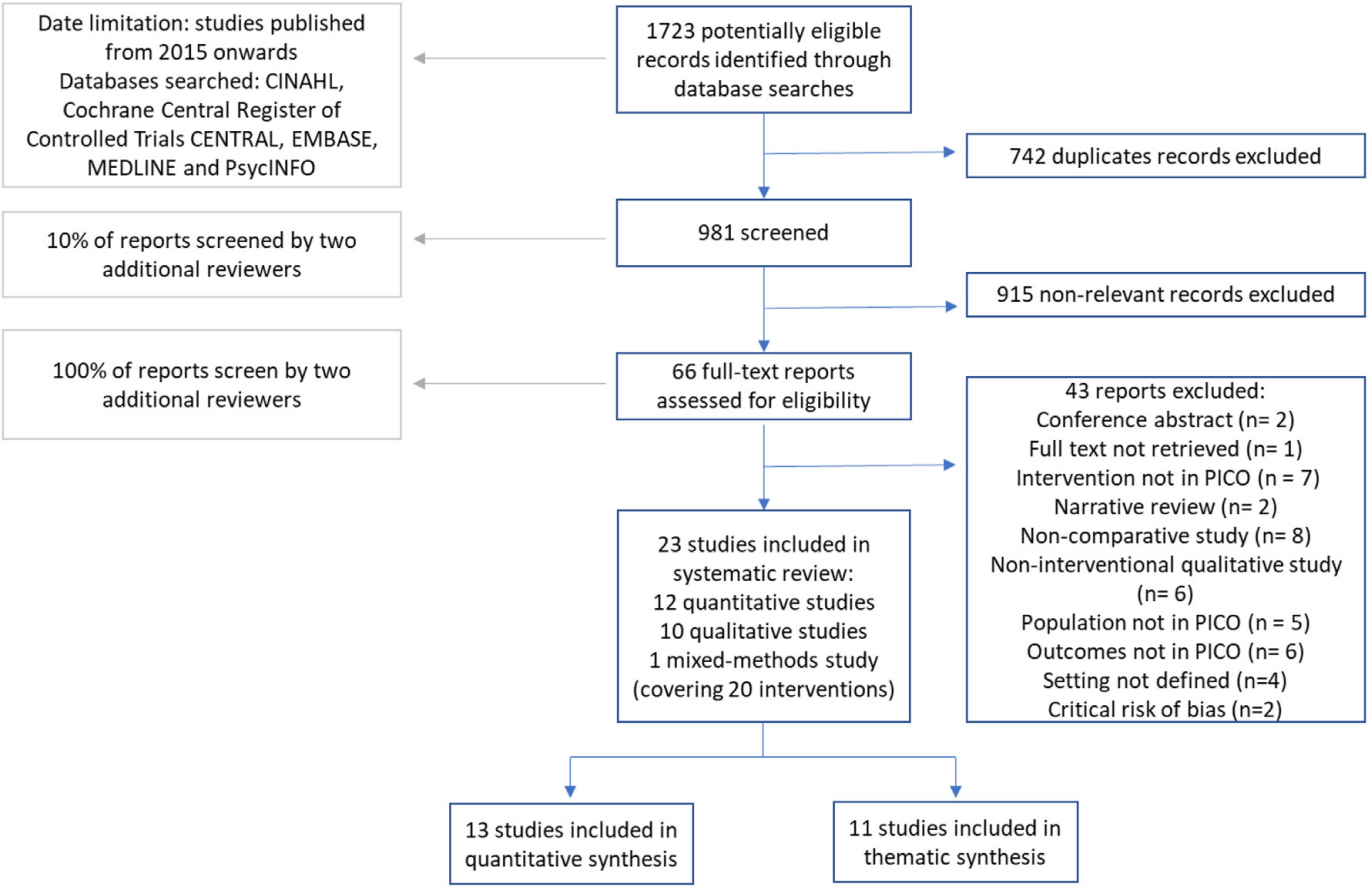
Study selection flow diagram.

1723 records were retrieved from database searches and 66 identified as potentially eligible from title and abstract screening after removing duplicates (Fig. 2). 23 studies were selected for inclusion (12 quantitative, 10 qualitative and one mixed method) covering 20 distinct interventions (three interventions contributed both a qualitative and a quantitative study).

Evidence from 13 studies contributed to the quantitative narrative synthesis and meta-analyses (Table 1): four RCTs^21^, ^22^, ^23^, ^24^; one quasi-randomised trial^25^; eight non-randomised studies, including five cohort study designs,^26^, ^27^, ^28^, ^29^, ^30^ two before-and-after study designs^31^^,^^32^ and one mixed methods study.^33^ Evidence from 10 qualitative studies^34^, ^35^, ^36^, ^37^, ^38^, ^39^, ^40^, ^41^, ^42^, ^43^ and one mixed methods study^33^ contributed to the thematic synthesis (Table 2). Three quantitative studies^24^^,^^30^^,^^31^ have ‘sibling’ qualitative studies^35^^,^^36^^,^^39^ pertaining to the same intervention; these are counted as one ‘study’ in the narrative description of study characteristics, but both are cited in the text.

**Table 1:** Characteristics of included quantitative studies.

Study reference	Country	Study design	Population	Intervention group^a^	Control group	Outcomes of interest	Risk of bias assessment
**Interventions providing material support only**							
**Durovni 2018** ^26^	Brazil	NRSI (retrospective cohort study)	• N = 495 • Adults (≥15 years) with a recorded TB outcome for ‘new’ TB case (not re-treatment case) • Registered to a clinic providing the Family Health Strategy programme (home visits and home or clinic-based DOT) in Rio de Janeiro • <4 years of schooling (proxy for poverty and eligibility for BFP cash-transfer intervention)	Material support • Financial: ‘Bolsa Familia’ (BFP) social protection cash transfer programme for poor families. Amount transferred dependent on income and family composition (monthly basic benefit of ∼USD 25) Sample • n = 96 • Received BFP intervention (as determined by payroll registers)	Standard care • Family Health Strategy programme (home visits and home or clinic-based DOT) Sample • n = 399 • Eligible for the BFP intervention, but did not receive it (as determined by payroll registers; further explanation for non-exposure not provided)	• Treatment success	Moderate
**Klein 2019** ^27^	Argentina	NRSI (prospective cohort study)	• N = 962 • Adults (≥18 years) with first diagnosis of pulmonary DS-TB • Registered at TB care facilities in high burden TB areas in Buenos Aires (BA)	Material support • Financial: monthly conditional cash-transfers (undisclosed amount; percentage of minimum wage) Sample • n = 377 • Eligible for cash-transfer if resident of BA for at least 2 years and not covered by any other social security system	Standard care • Clinic-based DOT or SAT Sample • n = 564 • Not eligible for cash-transfer as per criteria	• Treatment success • LTFU	Serious
**Samuel 2016** ^28^	India	NRSI (retrospective cohort study)	• N = 573 • Adults with recorded outcomes for first diagnosis or previously treated pulmonary DS-TB • Living below the poverty line (monthly income < USD 46)	Material support • Nutritional: monthly allocation of rice and lentils for up to 60–90 days during care (USD 10 per month) Sample • n = 173 • All patients who received nutritional support from two treatment units implementing the intervention	Standard care • DOT provided by NTP (setting unclear) Sample • n = 400 • Every other patient on a list of patients from two treatment units not implementing the intervention (located close to the implementing treatment units)	• Treatment success • Treatment failure • Death • LTFU	Serious
**Ukwaja 2017** ^31^	Nigeria	NRSI (prospective before and after study)	• N = 294 • Adults with first diagnosis of pulmonary DS-TB • Registered to a large outpatient TB facility in rural area with high prevalence of poverty	Material support • Financial: monthly cash incentives (USD 15) conditional on attending the clinic for DOT for 6 months Sample • n = 121 • All patients registered in 3-month post-intervention period	Standard care • Clinic-based DOT Sample • n = 173 • All patients registered in 3-month pre-intervention period	• Treatment success	Serious
**Interventions providing psychological-based support only**							
**Kaplan 2016** ^32^	South Africa	NRSI (before and after study)	• N = 23,210 • Adults with DS-TB and DR-TB with a recorded outcome reported 3–9 months after intervention initiation • Received care at outpatient TB clinics in Cape Town	Psychological-based support • Health education: delivered by trained lay community care workers (number and duration of sessions unknown) DOT • 2 weeks of clinic-based DOT, followed by weekly home visits by a community healthcare worker Sample • n = 11,314 • All patients starting TB treatment in 6-month post-intervention period	Standard care • Clinic-based DOT Sample • n = 11,896 • All patients starting TB treatment in 6-months pre-intervention period	• Treatment success • Treatment failure • LTFU • Death	Serious
**Khachadourian 2020** ^21^	Armenia	Cluster-RCT	• N = 385 • Adults (≥18 years) with DS-TB • Initiating continuation phase TB care and with recorded TB outcomes	Psychological-based support • Health education and counselling: one session after intensive phase care and before starting SAT • Adherence support • Daily SMS reminders and daily phone calls to family treatment supporter SAT/DOT • SAT for intervention group (supported by family member) with weekly visits to an outpatient facility Sample • n = 187	Standard care • Clinic-based DOT Sample • n = 198	• Treatment success • Treatment failure • Death • LTFU	Moderate
**Muller 2019** ^22^	Brazil	RCT	• N = 169 • Adults (≥18 years) initiating care for any newly diagnosed TB • In outpatient facilities in Brazil (following discharge from hospital)	Psychological-based support • Health education and counselling: patient given educational materials and one counselling session provided on discharge from hospital • Adherence support • Monthly phone calls from healthcare worker and contact with primary healthcare clinic every 3 months Sample • n = 187	Standard care • Clinic-based DOT or SAT Sample • n = 89	• Treatment success • Treatment failure • Death • LTFU	• Low
**Tola 2016** ^23^	Ethiopia	Cluster RCT	• N = 698 • Adults (>17 years) with TB (type not specified) • In TB care for 2 months at 30 urban health facilities in Addis Ababa	Psychological-based support • Health education and counselling: 7 sessions of 30 min during TB care Sample • n = 368	Standard care • Clinic-based DOT Sample • n = 330	• LTFU	Moderate
**Interventions providing psychosocial support (material and psychological-based support)**							
**Bhatt 2019** ^29^	India	NRSI (retrospective cohort study)	• N = 123 • Adults with DR-TB with recorded treatment outcomes from outpatient TB clinics	Material support • Nutritional: monthly food packages and supplements (based on need) • Financial: monthly cash handouts and transport reimbursement (based on need) Psychological-based support • Counselling: home visits by healthcare worker and peer-support meetings (no further detail provided) Sample • n = 60 • Patients deemed by healthcare workers in need of socioeconomic support (judgement criteria not defined) • Received support package for >3 months	Standard care • Clinic-based DOT (as per NTP guidelines) Sample • n = 63 • Patients not deemed in need of socioeconomic support, or; • Patients deemed in need of socioeconomic support who were transferred to another clinic before receiving support, or; • Patients who received support for <3 months	• Treatment success • Treatment failure • Death • LTFU	Serious
**Skiles 2018** ^30^	Ukraine	NRSI (retrospective cohort study)	• N = 708 • Adults with TB who had completed intensive phase treatment, initiated continuation therapy, and had a recorded treatment outcome • Considered high-risk for default (as defined by study authors) • Living in regions with high TB caseloads	Material support • Financial: vouchers for transportation and cash for other necessities (based on need) • Nutritional: food packages (based on need) Psychological-based support • Counselling and career support (based on need) DOT • Home- and clinic-based DOT (routine implementation varied by region) Sample • n = 397 • Sampled from a list of people enrolled in the intervention	Standard care • Not defined Sample • n = 311 • Matched to intervention participants (facility receiving therapy; start date for continuation therapy)	• Treatment success • LTFU	Moderate
**Taneja 2017** ^25^	India	RCT (Quasi-experimental trial)	• N = 100 • Adults diagnosed and being treated for MDR-TB with a treatment duration >6 months. • Patients with any form of disability, comorbidity, or pregnancy, were excluded	Material support • Nutritional: home-based (based on need; no further information) Psychological-based support • Health education and counselling: home based (based on need; no further information) Integrated care • Referrals, rehabilitation, career advice Sample • n = 50 • Patients in one of the two clinics (no further information provided)	Standard care • Clinic-based DOT (as per NTP guidelines) Sample • n = 50 • Patients in the other of the two clinics (no further information provided)	• Treatment success • LTFU	Moderate
**Wingfield 2017** ^24^	Peru	RCT	• N = 282 • People with TB initiating care through the Peruvian NTP, living in an area with a high prevalence of poverty	Material support • Financial: conditional cash transfers (≤ USD 230 in total) given throughout treatment Psychological-based support • Health education and counselling: one home-based visit after commenced treatment on TB and household finances; monthly community participatory peer-support meetings Sample • n = 135	Standard care • Clinic-based DOT (as per the Peruvian NTP) Sample • n = 147	• Treatment success	Moderate
**Yin 2018**^33^	China	Mixed methods study NRSI (retrospective cohort study) Qualitative (in-depth interviews conducted 2–5 years after TB care ended)	• N = 218 • People with MDR-TB initiating care through the Global Fund project • Living in poverty (subjectively judged by healthcare workers) • N = 20 for qualitative analysis (n = 10 people with MDR-TB, n = 10 treatment supporters)	Material support • Financial and nutritional: transportation vouchers (USD 10 per month) and food vouchers (USD 10 per month) for people living in poverty Psychological-based support • Health education: monthly sessions by family member or healthcare worker Sample • n = 100 (financial support) • n = 44 (health education)	Standard care • Clinic-based DOT or SAT	• Treatment success	Serious

Abbreviations: DOT (directly observed therapy); DR-TB (drug-resistant TB); DS-TB (drug-sensitive TB); MDR-TB (multi-drug resistant TB); NRSI (non-randomised study of an intervention); NTP (national TB programme); RCT (randomised controlled trial); SAT (self-administered therapy); USD (US Dollars).

a Unless otherwise stated, intervention group received standard of care as a co-intervention.

**Table 2:** Characteristics of included qualitative studies.

Study reference	Country	Aim	Sample population	Intervention	Data collection methods	Analysis methods	Risk of bias
**Interventions providing material support only**							
**Orlandi 2019**^40^	Brazil	To explore how social incentives work for improving adherence to TB care	• Primary healthcare workers in urban health facility caring for people with TB N = 86	Material support • Nutritional: snacks, food vouchers at clinic visits • Financial support: transportation vouchers provided at clinic visits and social protection measures for poverty alleviation DOT • Clinic-based DOT	SSIsconducted during TB care	Thematic framework analysis	Moderate
**Ukwaja 2017**^41^ *Sibling of*^31^	Nigeria	To explore the effectiveness, acceptability and feasibility of a financial incentive intervention	• People with TB (n = 103) and healthcare workers (n = 10) at large outpatient TB facility in rural area with high prevalence of poverty N = 113	Material support • Financial: monthly financial incentives (USD 15) conditional on attending the clinic for DOT for 6 months of care DOT • Clinic-based DOT	SSIs and FGDs conducted during final month of intervention	Thematic analysis	Low
**Interventions providing psychological-based support only**							
**Horter 2020**^43^	Uzbekistan	To explore the effectiveness and acceptability of a people-centered care approach	• People with MDR-TB who had completed short-course regimen (n = 24) • MDR-TB healthcare workers (n = 20) N = 44	Psychological-based support • Counselling: focusing on providing information about TB and guided by a shared decision- making approach DOT DOT • Clinic-based DOT and home-based DOT for people not able to travel to clinics	SSIs and FGDs conducted during TB care	Thematic analysis	Low
**Snyman 2018**^37^	South Africa	To explore the effectiveness and acceptability of a people-centered care approach	• People with DR-TB with a previous history of treatment interruption (n = 7) • TB support workers (n = 5), healthcare workers (n = 13) and programme coordinators (n = 3) N = 28	Psychological-based support • Counselling: home-based provided by trained community treatment supporter who previously had TB, with follow-up visits and calls provided as needed. Family meetings and support forums. Integrated care • Referrals to social workers, substance use centres or short-term inpatient care DOT • Clinic-based DOT or SAT by community healthcare worker	SSIs and FGD conducted during TB care, 4 years after initiation of intervention	Thematic network approach	Moderate
**Walker 2018**^38^	Nepal	To explore the feasibility and acceptability of the different components of a psychological-based support intervention	• People with MDR-TB (n = 5) • MDR-TB healthcare workers at two urban MDR-TB outpatient clinics (n = 2) N = 7	Psychological-based support • Health education: materials provided at the start of care to the person with TB and family members • Counselling: for people with depression (following a monthly screening), 8 sessions or until depression score reduced below a threshold and additional peer-support group counselling DOT • Not reported in study (assumed provided under Nepal's National TB Control Programme as standard of care)	SSIs conducted at month 9 and final month of intervention during TB care; reflective diaries and field visit reports	Thematic framework analysis	Moderate
**Interventions providing psychosocial support (material and psychological-based support)**							
**Burtscher 2020**^41^	Eswatini	To explore the effectiveness, feasibility and acceptability of the different components of a psychosocial support intervention within a community-based model of care	• People with DR-TB (n = 9) • Community treatment supporters (n = 11) and family members of people with DR-TB (n = 9) N = 29	Material support • Financial and nutritional: travel allowances and food packages Psychological-based support • Counselling: home-based with trained community treatment supporter and community TB nurse DOT • Home-based DOT by trained community treatment supporter	SSIs, paired interviews (with family members in patients' homes) and FGDs during TB care	Thematic analysis	Low
**Charyeva 2019**^39^ *Sibling of*^30^	Ukraine	To explore which components of a psychosocial support intervention worked	• People with TB high-risk for treatment default (n = 21) • TB healthcare workers (n = 11) and programme coordinators (n = 4) N = 36	Material support • Financial: vouchers for transportation and cash for other necessities (based on need) • Nutritional: food packages (based on need) Psychological-based support • Counselling and career support (based on need) DOT • Home-based DOT	SSIs conducted during TB care	Thematic analysis	Moderate
**Davtyan 2015**^a^^,^^34^	India	To explore the effectiveness and acceptability of the different components of a psychosocial support intervention	• People with DS-TB and DR-TB (n = 20) • TB healthcare workers (n = 20) N = 40	Financial and nutritional support • Transportation reimbursed • Food basket and hygiene package provided (once per month) Health education • No further details provided DOT • Clinic-based DOT	SSIs conducted after completion of care	Thematic analysis	High
**George 2020**^42^	India	To map the psychosocial support provided in different districts and explore their effectiveness, acceptability and feasibility	• People with TB who successfully completed TB care • TB healthcare workers N = not reported	Financial and nutritional support • Provided by referral to local social support schemes • Transportation allowance Psychological-based support • Counselling provided by referral to local social support schemes and peer-support via TB survivor programme Integrated care • Referrals to social support schemes and local services DOT • Method dependent on district	SSIs and FGDs conducted after TB care	Thematic analysis	Moderate
**Wingfield 2015**^35^ *Sibling of*^24^	Peru	To explore the acceptability of the different components of a psychosocial support intervention	• People with TB in care administered by the Peruvian National TBProgramme • Programme managers and civil society groups N = not reported	Material support • Financial: conditional cash transfers (≤USD 230 in total) Psychological-based support • Health education and counselling: home-based with a community healthcare worker and participatory peer-support community meetings DOT • Home-based	SSIs and FGDs	Unclear	High

Abbreviations: DOT (directly observed therapy); DR-TB (drug-resistant TB); DS-TB (drug-sensitive TB); FGDs (focus group discussions); MDR-TB (multi-drug resistant TB); SAT (self-administered therapy); SSIs (semi-structured interviews); USD (US Dollars).

a Davtytan 2015 is a mixed methods study, however only the qualitative data was used for this review due to the critical risk of bias of the quantitative data.

Risk of bias ranged from low to moderate for RCTs and the quasi-randomised trial: the main concerns were due to lack of blinding of participants and personnel due to the nature of the intervention (Supplementary Table S4). For NRSIs, the overall risk of bias ranged from moderate to serious, with the most common concerns due to confounding (unmeasured or unaccounted for in analytical approach), unclear classification of interventions, and missing data (Supplementary Table S5. Two studies were excluded following risk of bias assessment due to critical risk of selection bias.^44^^,^^45^ The quality of qualitative studies ranged from low to high (Supplementary Table S6).

Studies were conducted in 13 countries across five of the six WHO world regions: four studies in the African region (Ethiopia, Eswatini, Nigeria and South Africa)^23^^,^^31^^,^^36^^,^^37^^,^^41^; five studies in the Region of the Americas (Argentina, Brazil and Peru)^22^^,^^24^^,^^26^^,^^27^^,^^35^^,^^40^; five studies in the South-East Asia Region (India and Nepal)^25^^,^^28^^,^^29^^,^^38^^,^^42^; four studies in the European Region (Armenia, Ukraine and Uzbekistan)^21^^,^^30^^,^^34^^,^^39^^,^^43^; and one study in the Western Pacific Region (China).^33^ Nine studies were conducted in countries with a high incidence of TB, TB and HIV and multi-drug resistant TB (MDR-TB)^23^^,^^25^^,^^28^^,^^29^^,^^31^^,^^32^^,^^36^^,^^37^^,^^42^; three studies in countries with a high incidence of TB and TB and HIV^22^^,^^26^^,^^40^; three studies in countries with a high incidence of MDR-TB^24^^,^^30^^,^^35^^,^^39^^,^^43^ and five studies in countries not considered high incidence,^21^^,^^27^^,^^34^^,^^38^^,^^41^ according to the WHO classification at the time of the study. Ten studies included participants with any type of active TB or did not specify^22^, ^23^, ^24^^,^^26^^,^^30^^,^^32^^,^^34^^,^^35^^,^^39^^,^^40^^,^^42^^,^^45^; seven studies included only people with drug-resistant TB (DR-TB) or MDR-TB^25^^,^^29^^,^^33^^,^^37^^,^^38^^,^^41^^,^^43^; and four studies included only people with drug-sensitive TB (DS-TB).^21^^,^^27^^,^^28^^,^^31^^,^^36^

Five studies evaluating material support interventions were included. Four interventions provided financial support, either as conditional cash transfers at clinic visits,^27^^,^^31^^,^^36^ transportation vouchers^33^ or cash transfers as part of the Bolsa Familia social protection programme (BFP).^26^ Other studies evaluating BFP were excluded due to insufficient information on the setting for standard care. One intervention comprised nutritional support only, providing monthly food packages.^28^ One study provided snacks and food vouchers at clinic visits, as well as transportation vouchers at clinic visits or unconditional cash-transfers for people living in poverty.^40^

Seven of the included studies evaluated psychological-based support interventions offering counselling and/or health education. One intervention provided health education from lay healthcare workers.^32^ Two interventions offered counselling sessions, one as part of a home-based intervention delivered by a trained community treatment peer-supporter^37^ and the other in the form of a shared decision-making model of care.^43^ Four interventions provided both education and counselling, ranging from a single session to multiple sessions.^21^, ^22^, ^23^^,^^38^

Seven studies evaluated psychosocial support interventions providing both material and psychological-based support: three provided transportation vouchers or transportation reimbursement, food packages and either home-based counselling with a treatment supporter^29^^,^^30^^,^^39^^,^^41^ or health education with a community healthcare worker^34^; one provided nutritional support and home-based health education^25^; one provided conditional cash transfers, health education and counselling, including participatory peer-support community meetings.^24^^,^^35^ One of these studies offered peer-support group counselling sessions in addition to individual counselling.^29^ One qualitative study evaluated a range of locally-implemented psychosocial support schemes available to patients with TB, including counselling, a peer-support programme, and prevention of out-of-pocket expenditure, for example via food packages and transportation allowances.^42^ One mixed methods study evaluated a psychosocial support intervention offering transportation vouchers, food vouchers for people living in poverty, and health education delivered monthly by healthcare workers and family members^33^: the quantitative analysis measured treatment outcomes associated with exposure to each intervention component separately.

### Quantitative synthesis

A summary of the quantitative synthesis can be found in Table 3. Forest plots are presented in the supplementary material (Supplementary Figs. S1–S4) and GRADE assessment of the quality of the evidence for outcomes included in meta-analyses can be found in Supplementary Tables S7–S10.

**Table 3:** Summary of quantitative results.

Intervention type	Outcomes	Study type (RCT or NRSI)	Number of participants	Relative effect OR (95% CI)	Contributing studies	Quality of the evidence (GRADE)^b^
**Material support**						
**Financial support**	Treatment success	NRSI	1933	2.11 (1.45–3.06)	27, 30, 32, 33	Low
	LTFU	NRSI	933	0.49 (0.34–0.73)	30	N/A (moderate risk of bias as per ROBINS-I)
**Nutritional support**	Treatment success	NRSI	573	2.80 (1.57–5.01)	26	N/A (serious risk of bias as per ROBINS-I)
	Treatment failure	NRSI	483	1.35 (0.48–3.77)	26	N/A (serious risk of bias as per ROBINS-I)
	Death	NRSI	572	0.60 (0.27–1.34)	26	N/A (serious risk of bias as per ROBINS-I)
	LTFU	NRSI	510	0.40 (0.05–3.19)	26	N/A (serious risk of bias as per ROBINS-I)
**Psychological-based support**						
**Health education**	Treatment success	NRSI	23,429	0.96 (0.89–1.02)	31, 33	Low
	Treatment failure	NRSI	23,917	1.33 (0.87–2.06)	31	N/A (moderate risk of bias as per ROBINS-I)
	Death	NRSI	23,016	0.93 (0.81–1.06)	31	N/A (moderate risk of bias as per ROBINS-I)
	LTFU	NRSI	23,230	1.02 (0.94–1.12)	31	N/A (moderate risk of bias as per ROBINS-I)
**Counselling and health education**^a^	Treatment success	RCT	554	1.28 (0.64–2.54)	23, 24	Low
	Treatment failure	RCT	339	1.17 (0.50–2.75)	23, 24	Low
	Death	RCT	556	1.00 (0.51–1.98)	23, 24	Low
	LTFU	RCT	827	0.63 (0.06–6.59)	21, 24	Low
**Psychosocial support**						
**Combination of material and psychological-based support**	Treatment success	RCT and quasi-randomised trial	352	1.80 (1.17–2.75)	22, 25	Low
		NRSI	831	3.01 (2.14–4.25)	28, 29	Low
		RCT, quasi-randomised trial and NRSI (overall estimate)	1183	2.46 (1.89–3.22)	22, 25, 28, 29	Low
	Treatment failure	RCT	282	0.36 (0.01–8.92)	22	N/A (moderate risk of bias as per RoB v2)
		NRSI	831	1.51 (0.28–8.07)	28, 29	Very low
		RCT and NRSI (overall estimate)	1113	1.20 (0.31–4.70)	22, 28, 29	Very low
	Death	RCT	275	1.00 (0.30–3.36)	22	N/A (moderate risk of bias as per RoB v2)
		NRSI	832	0.31 (0.16–0.59)	28, 29	Low
		RCT and NRSI (overall estimate)	1107	0.43 (0.19–0.95)	22, 28, 29	Very low
	LTFU	RCT, quasi-randomised trial	352	0.76 (0.44–1.34)	22, 25	Very low
		NRSI	819	0.13 (0.06–0.28)	28, 299	Low
		RCT, quasi-randomised trial and NRSI (overall estimate)	1171	0.30 (0.10–0.92)	22, 25, 28, 29	Very low

Abbreviations: OR (odds ratio); CI (confidence interval); LTFU (loss-to-follow-up); NRSI (non-randomised studies of interventions) OR (odds ratio); RCT (randomised control trial).

a Studies evaluating interventions providing both counselling and health education were meta-analysed as no interventions providing only counselling were included in the review.

b N/A denotes where GRADE was not conducted as only one study provided evidence for the outcome.

#### Material support - financial and nutritional support

There was low quality evidence for a benefit of financial support interventions on: treatment success (OR 2.11, 95% CI 1.45–3.06; 1933 people; four NRSIs, I^2^ = 54%) and one study reported a significant reduction in LTFU for a conditional cash transfer intervention.^27^ One study which provided a monthly nutritional support package for people with DS-TB during care reported a benefit on treatment success, but not on treatment failure, death or LTFU,^28^ however this study was at serious risk of bias.

#### Psychological-based support - health education and counselling

There was low quality evidence from two RCTs that combined health education and counselling interventions had no effect on: treatment success (OR 1.28 95% CI 0.64–2.54; 554 people; two RCTs; I^2^ = 48%*),* treatment failure (OR 1.17 95% CI 0.50–2.75; 239 people; 2 studies; I^2^ = 9%)*,* death (OR 1.00 95% CI 0.51–1.98; 556 people; two studies; I^2^ = 0%) or LTFU (OR 0.63 95% CI 0.06–6.59; 827 people; two studies; I^2^ = 89%). One of these studies achieved treatment success of over 90% in both groups, however, the study population was restricted to people with DS-TB who had successfully completed the intensive phase of care, introducing a selection bias towards participants more likely to be cured and/or complete care.^21^ There was low to very low quality evidence that interventions providing health education alone had no effect on treatment success (OR 0.96 95% CI 0.89–1.02; 23,429 people; two studies; I^2^ = 0%) and one large before-and-after study reported no effect of health education on treatment failure, death or LTFU.^32^

#### Interventions combining material and psychological-based support

RCTs and NRSIs contributed data to the meta-analyses on treatment outcomes for combined psychosocial support interventions. Effect estimates by study design (RCT or NRSI) and overall effect estimates are presented in Table 3. There was low quality evidence that interventions providing both material and psychological-based support had a benefit on treatment success, with a larger benefit in the NRSI subgroup (OR 3.01, 95% CI 2.14–4.25, 2 studies, 831 people, I^2^ = 0%) compared to the RCT subgroup (OR 1.80, 95% CI 1.17–2.75, 2 studies, 352 people, I^2^ = 8%). One of the included RCTs which evaluated a financial support and community-based psychosocial intervention in Peru reported higher rates of treatment success for people from ‘poorer households’ than ‘less poor’ households, providing some evidence of ‘poverty-sensitivity’, however subgroup analyses were not powered to detect significant differences.^24^

There were differences by study design in the evidence contributing to the outcomes of death and LTFU. Two NRSIs provided very low quality evidence of a reduction in deaths (OR 0.31, 95% CI 0.16–0.59, 2 studies, 832 people, I^2^ = 11%), however one RCT provided evidence of no effect on deaths. For LTFU, two RCTs provided very low quality evidence of no effect (OR 0.76 95% CI 0.44–1.34, 2 studies, 352, I^2^ = 0%) whereas 2 NRSIs provided low quality evidence of a benefit (OR 0.13 95% CI 0.06–0.28, 2 studies, 819, I^2^ = 0%). There was very low quality evidence of no effect on treatment failure, with imprecision caused by low event rates for both RCTs and NRSI subgroups.

### Qualitative synthesis

Thirteen descriptive themes emerging from the thematic analysis associated with intervention components were categorised into mechanisms of action or intervention barriers (Table 4). Mechanisms of action themes were organised according to the SEM framework domains: individual, community and interpersonal, and structural. GRADE assessment of themes can be found in the supplementary material (Supplementary Table S11).

**Table 4:** Summary of qualitative results.

	Descriptive theme	Intervention component/active ingredient	Contributing Studies	Quality of the evidence (GRADE)
**Mechanisms of action**				
**Individual**	Improved access to treatment	• Financial support • Community-based support	36, 37, 39, 40, 41	Moderate
	Knowledge fosters autonomy	• Counselling • Health education	34, 37, 38, 39, 43	Moderate
	Improved mental health	• Counselling	38	Low
	Convenient care and flexible delivery	• Community-based support • Therapeutic relationship	39, 41	High
**Community and interpersonal**	Connectedness and optimism	• Peer support • Therapeutic relationship	35, 37, 38, 39, 40, 41, 42	Moderate
	Addressing material needs	• Financial support • Nutritional support	34, 36, 40	Low
**Structural**	Addressing TB-related stigma	• Community-based support • Therapeutic relationship	33, 35, 36, 41	Moderate
	Economic empowerment	• Financial support	35, 36, 40	Low
**Multi-level**	Multi-dimensional and multi-agency support	• Psychosocial interventions	34, 35	Low
	Patient-centered care	• Therapeutic relationship	36, 37, 38, 39, 40, 41	Moderate
**Intervention barriers**				
	Inadequate or inappropriate support	• Financial support • Nutritional support	33, 34, 35, 40, 42	Moderate
	Implementation delays	• Financial support	35, 42	Low
	Resource constraints	• Counselling • Nutritional support	38, 42	Low

In addition to the intervention components included in the quantitative analysis, community-based care (care delivered in the person's home or community), the therapeutic relationship between the person and their care team, and peer-support, emerged as active components of interventions and were included in the qualitative synthesis.

#### Individual level mechanisms

We identified four individual level mechanisms supporting improved access to, and engagement with, care: *‘improved access to care’, ‘convenient care and flexible delivery’, ‘knowledge fosters autonomy’*, and *‘improved mental health’.*

Financial support and community-based care mapped onto the theme *‘improved access to treatment’* as both eliminated the financial transportation barrier which enabled people with TB living in poverty to reach the health facility:

*“The money has been assisting me to come and collect my drugs. When there is no money, I cannot come… ”*

[person with TB, female, Nigeria]^36^

The theme *‘convenient care and flexible delivery’* describes the value people with TB placed in community-based models of care, noting that receiving care in their own homes at times suiting them allowed better management of side effects and to “*continue their everyday life”*^41^:

*“Everything is so simple that they bring it to you, you take it, and continue on with your activities”*

[person with TB, male, Ukraine]^39^

There was some evidence that the minimisation of everyday disruption over the duration of care was particularly important for people with MDR-TB.

While the quantitative synthesis did not find an effect of psychological-based support on treatment outcomes, qualitative data reveal how counselling and health education were associated with the theme *‘knowledge fosters autonomy*’. The majority of evidence contributing to this theme was derived from interventions for people with MDR-TB who found that receiving information about TB or talking about it with a counsellor enabled them to make informed decisions,^37^ promoting a sense of agency and improving motivation:

*“Having more information and understanding relating to MDR-TB and treatment appeared to support individuals having a sense of ownership over their health and treatment-taking”*

[primary author interpretation, study conducted in Uzbekistan]^43^

There was rich data from one study^42^ that counselling and health education *‘improved mental health’* for people with MDR-TB by helping them to better manage their mental health comorbidities, such as anxiety and depression:

*“Most of the patients found the information materials (particularly the pictures) helpful to understand MDR-TB and its management better, which reduced their mental stress”*

[primary author interpretation, study conducted in Nepal]^38^

#### Community and interpersonal level mechanisms

We identified two themes describing mechanisms of action at the community and interpersonal level: *‘addressing material needs’* and *‘connectedness and optimism’*.

By *‘addressing material needs’,* financial and nutritional support helped to relieve the wider socioeconomic pressures exacerbated by TB*.* Material support became a *“means of livelihood”* [healthcare worker, Brazil] (Orlandi 2019) for people with TB and in turn this helped to *“improve the person's, the family's quality of life”* [healthcare worker, Brazil]^40^:

*“In addition to helping with my transportation, this money helped reduce and relieve my sister from expenses she has been doing with her money for me”*

[person with TB, female, Nigeria]^36^

A theme associated with therapeutic relationships and peer-support was *‘connectedness and optimism’.* Peer-support was included in five studies in the form of group counselling, participatory community meetings, or deploying people who had recovered from TB as peer counsellors: these interventions allowed people to share their experiences, which in turn created a sense of *“solidarity and camaraderie”* [family member of a person with TB, Peru].^35^

People with TB found talking to people who had recovered from the disease to be comforting and encouraging, and a powerful motivator for continuing with care:

*“On seeing such people, they will realize that they too can survive if they take medicines. They get motivated and they go on completing their course”*

[person with TB, female, Nepal]^38^

Encouragement from healthcare workers fostered optimism and continued engagement with care:

*“[…] it’s my treatment supporter who has the big support that I have the energy to continue to take my treatment”*

[person with TB, male, Eswatini]^41^

For some people with TB who experienced social isolation, having someone who listened and encouraged them was particularly valued:

*“We sometimes sit down and talk about life and what stresses him. He treats me like his mother”*

[healthcare worker, female, Eswatini]^41^

#### Structural level mechanisms

We identified two themes describing mechanisms of action at the structural level: *‘economic empowerment’* and *‘addressing TB-related stigma’*.

Financial support acted through the mechanism of *‘economic empowerment’*, particularly for women and other vulnerable groups who were not normally “*financial decision makers”* [programme manager, Peru).^35^ The act of opening a bank account or receiving regular cash transfers could be transformative in terms of how people with TB perceived their life with the disease, with some expressing they felt more able to discuss their concerns about their disease with healthcare workers and families. There was evidence that financial independence, especially amongst marginalised groups, can increase agency in other aspects of life, including capacity to make health decisions and engage in care.

There was limited and conflicting evidence that community-based care and therapeutic relationships helped people with TB to engage with care through *‘addressing TB-related stigma’.* Community-based care delivered by a trusted healthcare worker reduced TB-related stigma for some people as they felt more able to talk about their illness than if they were at a clinic. Furthermore, it improved the perception of TB within the community as a result of people with TB being seen to continue their ‘normal’ life and get well. In contrast, there was evidence from one low quality study that regular home-visits from healthcare workers perpetuated anticipated stigma in some settings:

*“They had better follow me up via phone call than visit me at home. I don’t want to let the neighbours know my disease”*

[person with TB, female, China]^33^

It was noted that community-based care may be inappropriate for some people, for example for those who do not have a fixed address.

#### Multi-level mechanisms

‘*Patient-centered care’* was a component of the therapeutic relationship identified in six studies, operating at multiple levels to promote better engagement. Skilled and compassionate healthcare workers took a holistic approach and provided tailored support, for example taking time to understand the individual's biopsychosocial determinants of TB and existing individual, interpersonal, and societal support structures.

A theme shared by two interventions providing psychosocial support was the positive impact of *‘multi-dimensional* support *and integrated care’,* where providing both material and psychological-based support was found to operate on multiple levels to facilitate engagement with care. There was limited evidence that a multi-sectoral approach strengthened reach and sustainability through the outsourcing of different components of support to existing local providers.

#### Intervention barriers

We developed three themes addressing barriers to interventions: *‘insufficient or inappropriate for specific groups’*, *‘resource constraints’, and ‘implementation delays’.* There was evidence that financial and nutritional support could *be ‘insufficient or inappropriate for specific groups’*, for example: bank transfers for people who were not familiar or comfortable with using an ATM (automatic transfer machine), such as elderly people; those without a permanent address; rural populations; or people who did not want to be identified. In some settings, financial support was not sufficient to cover indirect costs of treatment or to support engagement, especially in the context of complex social marginalisation such as substance use. Nutritional support was not appropriate for some people who instead wanted the flexibility of financial support and to spend their money according to their current needs.

One study conducted in India indicated that *‘resource constraints’* was a major barrier to interventions associated with nutritional support, due to problems of ensuring the sustainability of food supply at the regional level.^42^ There was evidence from one study that a lack of sufficiently trained healthcare workers to deliver counselling sessions was an intervention barrier.^38^

Two studies highlighted *‘implementation delays’* as a barrier associated with cash transfer interventions.^35^^,^^42^ Rather than serving as an incentive for engagement with care, study authors described that people with TB found cash transfers stressful and demoralising if they were not received on time due to implementation issues, particularly for people who relied on such financial support to access care and support their dependents.

### Mixed methods integration: middle-range theories

A convergence coding matrix was used to explore the level of agreement between qualitative and quantitative syntheses and to generate three middle-range theories: effective interventions provide multi-dimensional support; psychological-based support is a transformative intervention component but is not sufficient to improve treatment outcomes; intervention delivery shapes a logic of care (Table 5). This process enriches the qualitative and quantitative data, maximising the potential of the mixed methods findings to generate explanatory and contextual middle-range theories which can be adapted to different settings.

**Table 5:** Convergence coding matrix of quantitative outcomes and qualitative themes.

Intervention	Quantitative outcomes, OR (95% CI)	Qualitative themes: Mechanism of action (SEM level)	Qualitative themes: intervention barrier	Convergence assessment
**Effective interventions provide multi-dimensional support and promote access to care**				
Financial support	• Treatment success (NRSI), 2.11 (1.45–3.06)	• Improved access to care (I) • Addressing material needs (Int) • Economic empowerment (S)	• Inadequate or inappropriate support • Implementation delays	Complementarity
Nutritional support	• Insufficient evidence to include in meta-analysis	• Addressing material needs (Int)	• Inadequate or inappropriate support • Resource constraints	Silence
Psychosocial interventions	• Treatment success (RCT), 1.80 (1.17–2.75) • Treatment success (NRSI), 3.01 (2.14–4.25) • Treatment success (overall), 2.46 (1.89–3.22)^a^ • Treatment failure (RCT), 0.36 (0.01–8.92) • Treatment failure (NRSI), 1.51 (0.28–8.07) • Treatment failure (overall), 1.20 (0.31–4.70)^a^ • Death (RCT), 1.00 (0.30, 3.36) • Death (NRSI), 0.31 (0.16–0.59) • Death (overall), 0.43 (0.19–0.95)^a^ • LTFU (RCT), 0.76 (0.44–1.34) • LTFU (NRSI), 0.13 (0.06–0.28) • LTFU (overall), 0.30 (0.10–0.92)^a^	• Multi-dimensional and multi-agency support (ML)	None identified	Complementarity
**Psychological-based support is transformative but is not sufficient to improve treatment outcomes (in the absence of improved access to care)**				
Health education	• Treatment success (NRSI), 0.96 (0.89–1.02)	• Knowledge fosters autonomy (I)	None identified	Dissonance
Counselling (and health education)	• Treatment success (RCT), 1.28 (0.64–2.54) • Treatment failure (RCT), 1.17 (0.50–2.75) • Death (RCT), 1.00 (0.51–1.98) • LTFU (RCT), 0.63 (0.06–6.59)	• Knowledge fosters autonomy (I) • Improved mental health (I)	• Resource constraints	Dissonance
Peer-support	Not reported	• Connectedness and optimism (Int/C)	None identified	Silence
**Intervention delivery shapes a logic of care**				
Community-based care	• Not reported	• Improved access to care (I) • Convenient care and flexible delivery (I) • Addressing TB-related stigma (S)	• Inadequate or inappropriate support	Silence
Therapeutic relationship	• Not reported	• Convenient care and flexible delivery (I) • Connectedness and optimism (Int) • Patient-centered care (ML)	None identified	Silence

Abbreviations: OR (odds ratio); CI (confidence interval); I (Individual); Int (Interpersonal); S (Societal); ML (multi-level); NRSI (non-randomised studies of interventions); RCT (randomised controlled study).

Findings from the quantitative and qualitative syntheses were integrated using a convergence coding matrix (Farmer 2006) to contextualize the findings in relation to different types of psychosocial support and detect the level of agreement: convergence (syntheses agree), complementarity (syntheses provide complementary information), dissonance (syntheses contradict one another) and silence (one synthesis provides limited or no evidence on this type of psychosocial support).

a Overall effect estimates combining RCTs and NRSIs.

#### Effective interventions provide multi-dimensional support

The thematic synthesis revealed that financial support, specifically cash-transfers, act at individual, interpersonal and structural levels to improve access and engagement with care, with the meta-analysis providing complementary evidence of a benefit for treatment success. Quantitative and qualitative findings relating to psychosocial interventions both demonstrated the importance of multi-dimensional support; of the studies included in this meta-analysis, only the smallest study which delivered home-based care^29^ did not provide financial support as a part of the psychosocial intervention. There was insufficient evidence to determine whether nutritional support alone improves treatment outcomes. Based on the patterns in the evidence, we hypothesise that a key tenet of effective interventions is the provision of multi-level support which facilitates improved access to care. The majority of the evidence contributing to this middle-range theory were from interventions specifically targeting patients who were socioeconomically vulnerable or conducted in settings with a high prevalence of poverty and it is not clear whether multi-level support is necessary for patients without these complex barriers to accessing care.

#### Psychological-based support is transformative but there is insufficient evidence that it improves treatment outcomes on its own

There was dissonance in the evidence for health education and counselling; while the thematic synthesis revealed that psychological-based intervention components act through individual and interpersonal mechanisms which transform mental health and social relationships, the meta-analyses reported no difference on treatment outcomes. This may be a symptom of psychological-based support acting on patient-reported outcomes which were not reported in this review, for example, health-related quality of life, depression and anxiety, stigma, and social isolation. However, this incongruence may also be methodological: only a small number of included studies provided psychological-based support only (most also provided material support) limiting the precision of the effect estimates. There were no quantitative data on the effectiveness of peer-support, likely because quantitative studies were not able to differentiate peer-support from counselling when both were offered, or studies did not measure peer-support in cases where it was delivered informally. Consistent with our first middle-range theory of the importance of multi-dimensional support, we conclude that conclude that there is currently insufficient evidence that psychological-based support on its own (without material support) improves treatment outcomes, although it is likely to have other important benefits for well-being.

#### Intervention delivery shapes a logic of care

Several themes illustrating the importance of how care is delivered (through community-based care and fostering a therapeutic relationship) emerged from the inductive thematic synthesis. Together these themes highlight that effective psychosocial support and treatment should be embedded within flexible practices, spaces and behaviours which are highly context-specific, and sensitive to the changing individual, interpersonal and societal needs and circumstances of the patient over the course of their care. This resonates with a ‘logic of care’, a term first described by medical anthropologist Anne-Marie Mol in her ethnography of people living with diabetes, which Mol contrasts with the hegemonic ‘logic of choice’ in current healthcare. Mol suggests that good care is a continuous process of applying logic to optimise life with a disease, basing actions and decisions on what a person needs at that time in a given situation.^46^

## Discussion

To our knowledge, this is the first systematic review to adopt a mixed methods convergent synthesis design to explore the effectiveness of psychosocial support interventions for people with TB. The strength of this approach lies in the contextualisation and enrichment of quantitative outcomes with the voices of people living with TB, healthcare workers, and others involved in delivering psychosocial support. It enables us to move beyond pre-determined quantitative endpoints and be attentive to what is important for the patient, unintended effects, mechanisms of action, and processes of implementation. The middle-range theories present guiding principles for psychosocial support interventions for people with TB: effective interventions provide multi-dimensional support; psychological-based support is transformative but there is insufficient evidence that it improves treatment outcomes on its own; intervention delivery shapes a logic of care.

Our results indicate that improving access to care is an essential mechanism underpinning effective interventions. Other systematic reviews have identified financial support as an effective component to improve TB treatment outcomes,^47^^,^^48^ however, to what extent outcomes were attributable to financial support was not clear and underlying mechanisms were not explored. We found that cash transfers are effective via multi-dimensional mechanisms, removing access barriers to care and fostering agency at a broader level, consequently improving engagement with care. In terms of implementation, attention is needed to ensure that financial support is timely, proportional to the needs of the individual and delivered via a suitable means. Where appropriate, cash-transfers should be considered as a foundational component of psychosocial support for people with TB, upon which other forms of support are layered, depending on need.

In addition to the nature of the support provided, how such interventions are delivered were found to be an important part of their mode of action. A strong therapeutic relationship and care delivered in the home or community act synergistically to provide a conduit through which to deliver general interventions via a ‘logic of care’, or a convenient, flexible, and people-centered approach. The role of the patient-provider bond within TB care has been highlighted in other studies.^49^ Training a sufficient number of healthcare workers to deliver psychosocial support can be challenging. Our study did not include interventions focusing on healthcare worker training, however, impact evaluations of lay TB healthcare worker training interventions in Malawi reported no impact on TB outcomes^50^ and a process evaluation identified several barriers to implementation, scalability and sustainability.^51^ Further research is needed to understand what works in terms of training, including who to train, to foster a therapeutic relationship at the heart of people-centered TB care.

We found insufficient evidence that psychological-based support on its own improves standard TB treatment outcomes, however, health education, counselling and peer-support can be valuable and transformative components of psychosocial interventions, particularly for MDR-TB patients and those with mental health comorbidities. The quality of the quantitative evidence was limited by the small number of studies included which only provided psychological-based support, with most studies providing health education and/or counselling alongside material support. This adds some context as to why there is firstly a paucity of evidence and secondly inconclusive evidence on the effectiveness of psychological-based support on standard TB outcomes.^19^^,^^52^

The findings of this review should be considered in light of its internal and external validity. We included both randomised and non-randomised studies of interventions. The risk of bias of NRSIs ranged from moderate to serious risk of bias, with a central concern being selection bias and insufficient data within the majority of studies to report adjusted odds ratios. Our approach to inclusion was informed by pragmatism owing to the nature of the evidence base and we have mitigated this risk of bias as far as possible by excluding studies at critical risk of bias in any domain; studies were excluded if control group populations were not eligible for the intervention and no methods to control for differences between groups at selection were used.

We acknowledge that the interventions studied are highly variable in both content and the systems in which they operate; this is a key feature of complex interventions. However, it is important for such interventions to be rigorously assessed; with transparent reporting of study design features and risk of bias, mixed methods systematic reviews allow for such data to be synthesised in order to build an evidence base that can inform policy and practice. Mixed methods reviews take a complexity perspective, with qualitative data giving an insight into how interventions are contingent on variations in design and context.^10^ An alternative approach, where studies are only pooled if standardised in contact and content, would likely result in a single study analysis, which would be of limited use to policy and guideline makers of complex interventions.

A limitation of the quantitative synthesis was the lack of patient-reported outcome measures which can be used to quantify how patients experience care. A core set of patient-reported outcome measures for people living with TB may increase inclusion of these measures within studies. All qualitative studies were cross-sectional, ranging in quality from low to high: there is a need for longitudinal qualitative research to explore how psychosocial support might influence access to and engagement with care over time. However, triangulation of qualitative findings and integration of quantitative and qualitative syntheses strengthens internal validity. Finally, this review was limited to English language studies, and we might have missed relevant evidence from studies published in other languages.

The generalisability of our findings to low incidence settings or high-income countries is limited as the majority of the evidence was from studies conducted in low- and middle-income countries with a high incidence of TB, or in settings with a high prevalence of poverty. We did not explore sustainability or how to successfully embed psychosocial support interventions within existing NTPs or integrate psychological-based support with existing services. Furthermore, we have not explored how psychosocial support interventions work in the context of health systems, politics, and culture; we strongly suggest that these structural level factors are taken into consideration when interpreting the findings. There is a need for further research to determine cost-effectiveness of psychosocial support interventions in different settings.

This review is timely in providing actionable insights to inform the design and implementation of locally-appropriate and people-centered psychosocial support. TB disproportionately affects individuals and communities living in low- and middle-income countries with biopsychosocial risk factors and requires a multi-dimensional approach to care. TB incidence has increased as a result of the COVID-19 pandemic, with health systems’ resources remaining constrained and a protracted detrimental impact on the psychosocial wellbeing of people with TB. This review updates the evidence of the effectiveness of psychosocial support interventions and presents an interpretation of the active components, mechanisms of action and considerations for successful implementation within NTPs.

## Contributors

CM, NF and MB conceptualised and designed the study. CM designed the search strategy and selection criteria. CM, ST and GS contributed to the screening, data extraction and quality assessment. CM synthesised the quantitative and qualitative data, with help from GS and NF andm ST. CM, NF, GS and ST accessed and verified the data. CM, NF and ST contributed to the integration of the mixed methods data and all authors contributed to the interpretation of the data. CM wrote the first draft of the paper, and all authors were involved reviewing and editing subsequent versions of the manuscript.

## Data sharing statement

Data will be available upon request from the corresponding author.

## Declaration of interests

ST has received consulting fees from Gilead Sciences and Sophia Forum. ST has received payment or honoraria for lectures, presentations, speakers bureaus, manuscript writing or educational events from Gilead Sciences and ViiV Healthcare. ST has a leadership or fiduciary role in Positively UK (Vice Chair of the Board of Trustees), Tommy's (Trustee), SWIFT (Trustee).
