# Supplementary material for: Psychosocial support interventions to improve treatment outcomes for people living with tuberculosis: a mixed methods systematic review and meta-analysis
Source: eClinicalMedicine. 2023 Jun 27;61:102057. doi: 10.1016/j.eclinm.2023.102057 (PMC10338299; doi:10.1016/j.eclinm.2023.102057)
Supplement: Supplementary Tables S1–S3 [file mmc1.docx]

**Contents**

[Supplementary Table 1: Treatment outcome definitions 2](#_Toc137995200)

[Supplementary Table 2. Eligibility criteria 3](#_Toc137995201)

[Supplementary Table 3: Search Strategy for MEDLINE 5](#_Toc137995202)

### Supplementary Table 1: Treatment outcome definitions

| Outcome | WHO definition |
| --- | --- |
| Treatment success | The sum of cured and treatment completed |
| Treatment completion | A TB patient who completed treatment without evidence of failure BUT with no record to show that sputum smear or culture results in the last month of treatment and on at least one previous occasion were negative, either because tests were not done or because results are unavailable. |
| Cure | A TB patient with bacteriologically confirmed TB at the beginning of treatment who was smear or culture negative in the last month of treatment and on at least one previous occasion |
| Lost to follow-up | Interruption of treatment for two months or more consecutively |

### Supplementary Table 2. Eligibility criteria

| PICO domain | Quantitative studies |
| --- | --- |
| Population | **Inclusion**: adults (16 years old and above) diagnosed with and enrolled for treatment for any clinical form of active TB, including those with HIV co-infection  **Exclusion**: children (below 16 years)*; people diagnosed with latent TB |
| Intervention | **Inclusion**: psychosocial support, defined as any psychological support (including, but not limited to: counselling sessions, peer- support or health education) or material support (including, but not limited to: financial incentives, transportation vouchers, food vouchers, food packages or supplements) or a combination thereof  **Exclusion**: variations of DOT or adherence interventions only, such as treatment ‘tracers’, reminder systems or digital monitors |
| Comparator | **Inclusion**: standard TB care (such as directly observed therapy) delivered in outpatient or ambulatory settings, such as primary health care facilities, TB clinics, community health posts or in the home  **Exclusion**: standard TB care (such as directly observed therapy) delivered in inpatient settings, such as hospitals |
| Outcomes | • Treatment success: the sum of people cured and completing treatment (main outcome)  • Cured: TB patient with bacteriologically confirmed TB at the beginning of treatment who was smear or culture negative in the last month of treatment and on at least one previous occasion  • Treatment completed: TB patient who completed treatment without evidence of failure but with no record to show that sputum smear or culture results in the last month of treatment and on at least one previous occasion were negative, either because tests were not done or because results are unavailable  • Treatment failure: TB patient whose sputum smear or culture is positive at month 5 for TB patients or month 8 for MDR/XDR-TB patients, or later, during treatment (main outcome)  • Lost to follow-up: TB patient whose treatment was interrupted for 2 consecutive months or more (main outcome)  • Died: A TB patient who dies for any reason during the course of treatment (main outcome)  • Patient-reported outcomes, including, but not limited to: quality of life, mental health outcomes (depression, anxiety), stigma, knowledge (of TB) (additional outcomes) |

| PICO domain | Qualitative studies |
| --- | --- |
| Population | **Inclusion**: adults (16 years old and above) diagnosed with and enrolled for treatment for any clinical form of active TB, including those with HIV co-infection, in addition to TB healthcare workers, treatment supporters, family members, programme managers or other relevant stakeholders  **Exclusion**: children (below 16 years old)*; people diagnosed with latent with TB |
| Intervention | **Inclusion**: psychosocial support, defined as any psychological support (including, but not limited to: counselling sessions, peer- support or health education) or material support (including, but not limited to: financial incentives, transportation vouchers, food vouchers, food packages or supplements) or a combination thereof  **Exclusion**: variations of DOT or adherence interventions only, such as treatment ‘tracers’, reminder systems or digital monitors |
| Comparator | Not required to have a control or comparator group |
| Outcomes | Lived experiences and perspectives of people living with and being treated for TB (main outcome); lived experiences and perspectives of TB healthcare workers, treatment supporters, family members, programme managers and other relevant stakeholders (additional outcomes) |

*include study if children are a sub-population of study

###

### Supplementary Table 3: Search Strategy for MEDLINE

| 1 | exp *Patient-Centered Care/ |
| --- | --- |
| 2 | exp Directly Observed Therapy/ |
| 3 | exp Primary Health Care/ |
| 4 | exp Tuberculosis/ |
| 5 | exp social support/ or psychosocial support systems/ |
| 6 | exp Ambulatory Care/ |
| 7 | *Counseling/ |
| 8 | exp social stigma/ |
| 9 | exp Mental Health/ |
| 10 | *"Quality of Life"/ |
| 11 | (stigma or depression or anxiety or knowledge).mp. [mp=title, abstract, original title, name of substance word, subject heading word, floating sub-heading word, keyword heading word, organism supplementary concept word, protocol supplementary concept word, rare disease supplementary concept word, unique identifier, synonyms] |
| 12 | (treatment adj2 (succes* or fail* or complet* or incomplet*)).mp. [mp=title, abstract, original title, name of substance word, subject heading word, floating sub-heading word, keyword heading word, organism supplementary concept word, protocol supplementary concept word, rare disease supplementary concept word, unique identifier, synonyms] |
| 13 | (psychosocial or counselling or voucher or transport* or education* or incentiv* or monetary).mp. [mp=title, abstract, original title, name of substance word, subject heading word, floating sub-heading word, keyword heading word, organism supplementary concept word, protocol supplementary concept word, rare disease supplementary concept word, unique identifier, synonyms] |
| 14 | (psychosocial or counselling or voucher or transport* or education* or incentiv* or monetary).mp. [mp=title, abstract, original title, name of substance word, subject heading word, floating sub-heading word, keyword heading word, organism supplementary concept word, protocol supplementary concept word, rare disease supplementary concept word, unique identifier, synonyms] |
| 15 | (food adj2 (package or supplement or basket or assistance)).mp. [mp=title, abstract, original title, name of substance word, subject heading word, floating sub-heading word, keyword heading word, organism supplementary concept word, protocol supplementary concept word, rare disease supplementary concept word, unique identifier, synonyms] |
| 16 | (cash adj2 transfer).mp. [mp=title, abstract, original title, name of substance word, subject heading word, floating sub-heading word, keyword heading word, organism supplementary concept word, protocol supplementary concept word, rare disease supplementary concept word, unique identifier, synonyms] |
| 17 | (support adj2 (emotional or social or education* or nutrition* or psychosocial or psychological or financial or food or peer or community or group or counselling or home or work or employ*)).mp. [mp=title, abstract, original title, name of substance word, subject heading word, floating sub-heading word, keyword heading word, organism supplementary concept word, protocol supplementary concept word, rare disease supplementary concept word, unique identifier, synonyms] |
| 18 | (tuberculosis or tb).mp. [mp=title, abstract, original title, name of substance word, subject heading word, floating sub-heading word, keyword heading word, organism supplementary concept word, protocol supplementary concept word, rare disease supplementary concept word, unique identifier, synonyms] |
| 19 | (patient-centered care or patient-centred care or people-centered care or people-centred care).mp. [mp=title, abstract, original title, name of substance word, subject heading word, floating sub-heading word, keyword heading word, organism supplementary concept word, protocol supplementary concept word, rare disease supplementary concept word, unique identifier, synonyms] |
| 20 | ((Ambulatory or community or decentrali* or outpatient or out-patient or primary) adj2 care).mp. [mp=title, abstract, original title, name of substance word, subject heading word, floating sub-heading word, keyword heading word, organism supplementary concept word, protocol supplementary concept word, rare disease supplementary concept word, unique identifier, synonyms] |
| 21 | 4 or 18 |
| 22 | 5 or 7 or 14 or 15 or 16 or 17 |
| 23 | 1 or 2 or 3 or 6 or 19 or 20 |
| 24 | 8 or 9 or 10 or 11 or 12 or 13 |
| 25 | 21 and 22 and 23 and 25 |
