# Supplementary material for: Psychosocial support interventions to improve treatment outcomes for people living with tuberculosis: a mixed methods systematic review and meta-analysis
Source: eClinicalMedicine. 2023 Jun 27;61:102057. doi: 10.1016/j.eclinm.2023.102057 (PMC10338299; doi:10.1016/j.eclinm.2023.102057)
Supplement: Supplementary Tables S4–S6 [file mmc2.docx]

**Contents**

[Supplementary Table 4: Risk of bias assessment for RCTs using RoB tool 2](#_Toc137995356)

[Supplementary Table 5: Risk of bias assessment for non-randomised studies using ROBINS-I 3](#_Toc137995357)

[Supplementary Table 6: CASP quality appraisal checklist for qualitative studies 6](#_Toc137995358)

### Supplementary Table 4: Risk of bias assessment for RCTs and quasi- experimental trial using RoB tool

| Study | Risk of bias domain | Rating | Support for judgement |
| --- | --- | --- | --- |
| Khachadourian 2020 | Random sequence generation | Low | Computer-assisted block randomisation to generate list of TBOCs for each arm |
|  | Allocation concealment | Some concerns | No mention of allocation concealment |
|  | Blinding of participants and personnel | Some concerns | Participants not blinded. Outcomes collected by health care workers at TB clinics, only non-clinical outcomes blinded. Potential contamination between arms. |
|  | Blinding of outcome assessment | Some concerns | Unclear if outcome assessment was blinded |
|  | Incomplete outcome data | Low | Per-protocol analysis presented alongside intention-to-treat as differential departure from allocation between arms. But intention to treat measures used in analysis |
|  | Selective reporting | Low | Protocol not available, but outcomes reported as expected |
|  | Other sources | Some concerns | Patients randomised after successful completion of intensive phase treatment likely to have introduced selection bias |
|  | Overall risk of bias | Some concerns/ moderate |  |
| Muller 2019 | Random sequence generation | Low | Details of block randomization described |
|  | Allocation concealment | Low | Sealed envelopes were used for allocation concealment |
|  | Blinding of participants and personnel | Some concerns | No mention, but unable to blind participants and personnel due to nature of intervention |
|  | Blinding of outcome assessment | Low | Outcome assessment blinded |
|  | Incomplete outcome data | Some concerns | 11 randomized participants died before receiving the intervention and were removed from analysis; unlikely to have introduced bias |
|  | Selective reporting | Low | Outcomes reported as expected in protocol |
|  | Other sources | Low | Baseline characteristics differ between intervention and control groups, but analysis adjusted for baseline differences |
|  | Overall risk of bias | Low |  |
| Taneja 2017 | Random sequence generation | Some concerns | Two centers chosen from a list of 20 in which a larger trial is being planned. No details on characteristics of centers |
|  | Allocation concealment | Some concerns | No mention of allocation concealment |
|  | Blinding of participants and personnel | Some concerns | No mention, but unable to blind participants and personnel due to nature of intervention |
|  | Blinding of outcome assessment | Some concerns | No mention of blinding of outcome assessors |
|  | Incomplete outcome data | Some concerns | Attrition bias due to only 38/50 patients in control and 32/50 in intervention available for follow-up outcome assessment. No clarification on this source of LTFU |
|  | Selective reporting | Low | Protocol not available, but outcomes reported as expected |
|  | Other sources | Unclear | No adjustment for confounding variables likely to affect outcome and most not measured i.e previous TB, other comorbdities i.e HIV or diabetes, income status (may be different between two treatment centers) |
|  | Overall risk of bias | Some concerns/ moderate |  |
| Tola 2016 | Random sequence generation | Some concerns | Simple random sampling used to randomise health centers to experimental arms |
|  | Allocation concealment | Unclear | No mention of allocation concealment |
|  | Blinding of participants and personnel | Some concerns | Not feasible to blind participants and personnel due to nature of the intervention |
|  | Blinding of outcome assessment | Unclear | No mention of blinding assessors |
|  | Incomplete outcome data | Low | Patients who died or were transferred out were not included in final analysis for non-adherence, but small percentages of patient population, so unlikely to introduce bias |
|  | Selective reporting | Unclear | Protocol not available and unclear why standard treatment outcomes such as 'cure' and 'completion' were not reported |
|  | Other sources | Unclear | Risk of reporting bias from healthcare workers and participants and HCWs implemented intervention and collected outcomes, possibly introducing social desirability bias |
|  | Overall risk of bias | Some concerns/ moderate |  |
| Wingfield 2017 | Random sequence generation | Low | Satisfactory method for randomisation used |
|  | Allocation concealment | Low | Satisfactory method for allocation concealment used |
|  | Blinding of participants and personnel | Some concerns | Not feasible to blind participants and personnel due to nature of the intervention |
|  | Blinding of outcome assessment | Some concerns | Unclear who collected the outcome and who assessed it |
|  | Incomplete outcome data | High | High percentage of patients LTFU (but similar between arms). Patients still taking treatment at 28 weeks (mostly MDR-TB patients) excluded from analysis as after follow-up (16% in intervention and 25% in control) |
|  | Selective reporting | Low | Protocol not available, but outcomes reported as expected |
|  | Other sources | Unclear | Mechanism of conditional cash transfer likely to have introduced bias |
|  | Overall risk of bias | Some concerns / moderate |  |

### Supplementary Table 5: Risk of bias assessment for non-randomised studies using ROBINS-I

| Study | Risk of bias domain | Rating | Support for judgement |
| --- | --- | --- | --- |
| **Bhatt 2019** | Bias due to confounding | Serious | Incomplete measurement of baseline confounding (HIV status); appropriate method to control for confounding not carried out |
|  | Bias in selection of participants into the study | Serious | Sub-set of the control group selected based on post-intervention variable (received support package for less than 3 months) |
|  | Bias in classification of interventions | Moderate | Intervention groups not clearly defined (‘dose’; frequency) |
|  | Bias due to deviations from intended interventions | Low | Deviations from intended intervention beyond usual practice not expected |
|  | Bias due to missing data | Moderate | All participants accounted for, but only those who had recorded treatment outcome were included (retrospectively) |
|  | Bias in measurement of outcomes | Low | Methods of outcome assessment comparable across groups; unknown if outcome assessors blinded to the intervention |
|  | Bias in selection of the reported result | Low | Negligible assessor judgment |
|  | Overall bias | Serious |  |
| **Durovni 2018** | Bias due to confounding | Moderate | Baseline confounding measured but not presented (review intervention groups used not primary intervention groups); adjustment for confounders carried out - direction of effect and assessment of significance did not differ from unadjusted estimates. |
|  | Bias in selection of participants into the study | Low | Participant selection based on variables observed before the intervention |
|  | Bias in classification of interventions | Low | Groups clearly defined at start of intervention |
|  | Bias due to deviations from intended interventions | Low | Deviations from intended intervention beyond usual practice not expected |
|  | Bias due to missing data | Low | All participants accounted for |
|  | Bias in measurement of outcomes | Low | Methods of outcome assessment comparable across groups; unknown if outcome assessors blinded to the intervention |
|  | Bias in selection of the reported result | Low | Negligible assessor judgment |
|  | Overall bias | Moderate |  |
| **Kaplan 2016** | Bias due to confounding | Serious | Significant differences in baseline confounding variables detected;; adjustment for confounders carried out - direction of effect and assessment of significance did not differ from unadjusted estimates. |
|  | Bias in selection of participants into the study | Low | Participant selection based on variables observed before the intervention |
|  | Bias in classification of interventions | Low | Groups clearly defined at start of intervention |
|  | Bias due to deviations from intended interventions | Low | Deviations from intended intervention beyond usual practice not expected |
|  | Bias due to missing data | Low | All participants accounted for |
|  | Bias in measurement of outcomes | Low | Methods of outcome assessment comparable across groups; unknown if outcome assessors blinded to the intervention |
|  | Bias in selection of the reported result | Low | Negligible assessor judgment |
|  | Overall bias | Serious |  |
| **Klein 2019** | Bias due to confounding | Serious | Significant differences in baseline confounding variables detected;; study adjustedt for confounders and matched control group participants to intervention group participants - direction of effect and assessment of significance did not differ from unadjusted estimates. |
|  | Bias in selection of participants into the study | Low | Participant selection based on variables observed before the intervention. |
|  | Bias in classification of interventions | Moderate | Groups clearly defined at start of intervention; timing and ‘dose’ received oof intervention not clear (only 20% of intervention group received the intervention during treatment) |
|  | Bias due to deviations from intended interventions | Low | Deviations from intended intervention beyond usual practice not expected |
|  | Bias due to missing data | Low | Nearly all participants accounted for |
|  | Bias in measurement of outcomes | Low | Methods of outcome assessment comparable across groups; unknown if outcome assessors blinded to the intervention |
|  | Bias in selection of the reported result | Low | Negligible assessor judgment |
|  | Overall bias | Serious |  |
| **Samuel 2016** | Bias due to confounding | Moderate | Incomplete measurement of baseline confounding (HIV); no baseline differences detected in measured variables (age, sex, treatment history). All participants living below the poverty line. |
|  | Bias in selection of participants into the study | Moderate | Participant selection based on variables observed before the intervention (quasi-random selection based on registration at one of two clinics)clinics geographically close, but no description of service-level characteristics of the two clinics. |
|  | Bias in classification of interventions | Moderate | Groups clearly defined at start of intervention; timing and ‘dose’ received of intervention not clear |
|  | Bias due to deviations from intended interventions | Low | Deviations from intended intervention beyond usual practice not expected |
|  | Bias due to missing data | Serious | Not all participants accounted for; Larger proportion of participants lost to follow-up in control group (10%) compared to intervention (1%) and no description of those lost |
|  | Bias in measurement of outcomes | Low | Methods of outcome assessment comparable across groups; unknown if outcome assessors blinded to the intervention |
|  | Bias in selection of the reported result | Low | Negligible assessor judgment |
|  | Overall bias | Serious |  |
| **Skiles 2018** | Bias due to confounding | Moderate | Baseline confounding variables sufficiently measured; control group participants matched to intervention group participants |
|  | Bias in selection of participants into the study | Low | Control group matching carried out (facility; date of initiation of continuation therapy; high-risk for treatment default) |
|  | Bias in classification of interventions | Moderate | Group definition ambiguous; insufficient information to ascertain why control group participants were unexposed; timing and ‘dose’ received of intervention not clear |
|  | Bias due to deviations from intended interventions | Low | Deviations from intended intervention beyond usual practice not expected |
|  | Bias due to missing data | Low | All participants accounted for |
|  | Bias in measurement of outcomes | Low | Methods of outcome assessment comparable across groups; unknown if outcome assessors blinded to the intervention |
|  | Bias in selection of the reported result | Low | Negligible assessor judgment |
|  | Overall bias | Moderate |  |
| **Ukwaja 2017a** | Bias due to confounding | Serious | Significant differences in baseline confounding variables detected; study adjustmed for confounders - direction of effect and assessment of significance did not differ from unadjusted estimates; possible time-varying confounding not accounted for |
|  | Bias in selection of participants into the study | Low | Participant selection based on variables observed before the intervention |
|  | Bias in classification of interventions | Moderate | Timing and ‘dose’ received of intervention not clearly defined |
|  | Bias due to deviations from intended interventions | Low | Deviations from intended intervention beyond usual practice not expected |
|  | Bias due to missing data | Moderate | Not all participants accounted for; follow up rate < 90% and no description of those lost |
|  | Bias in measurement of outcomes | Low | Methods of outcome assessment comparable across groups; unknown if outcome assessors blinded to the intervention |
|  | Bias in selection of the reported result | Low | Negligible assessor judgment |
|  | Overall bias | Serious |  |
| **Yin 2018** | Bias due to confounding | Serious | Unmeasured baseline confounding (between intervention groups) |
|  | Bias in selection of participants into the study | Moderate | Insufficient detail |
|  | Bias in classification of interventions | Serious | Groups not clearly defined; exposure based on self-report |
|  | Bias due to deviations from intended interventions | Low | Deviations from intended intervention beyond usual practice not expected |
|  | Bias due to missing data | Moderate | Not all participants accounted for; participants lost to follow-up excluded from the analysis (follow-up rate < 90%; 15% LTFU) |
|  | Bias in measurement of outcomes | Low | Methods of outcome assessment comparable across groups; unknown if outcome assessors blinded to the intervention |
|  | Bias in selection of the reported result | Low | Negligible assessor judgment |
|  | Overall bias | Serious |  |

### Supplementary Table 6: CASP quality appraisal checklist for qualitative studies

| Study | Quality appraisal domain | Rating (Yes/ No/ Can’t tell) | Support for judgement |
| --- | --- | --- | --- |
| Burtscher 2020 | 1. Was there a clear statement of the aims of the research? | Yes | Research aim stated |
|  | 2. Is a qualitative methodology appropriate? | Yes | Study aims to explore patients’ experiences and perceptions of TB services |
|  | 3. Was the research design appropriate to address the aims of the research? | Yes | Research design justified and linked to aims |
|  | 4. Was the recruitment strategy appropriate to the aims of the research? | Yes | Purposive recruitment strategy described |
|  | 5. Was the data collected in a way that addressed the research issue? | Yes | Methods and setting of data collection discussed and justified; data saturation discussed |
|  | 6. Has the relationship between researcher and participants been adequately considered? | No | No statement |
|  | 7. Have ethical issues been taken into consideration? | Yes | Ethical clearance obtained |
|  | 8. Was the data analysis sufficiently rigorous? | Yes | In-depth discussion of analysis process; derivation of themes is explicit; methods to minimize bias described |
|  | 9. Is there a clear statement of findings? | Yes | Findings are explicit and relevant data presented to support findings |
|  | 10. How valuable is the research? | Yes | Findings discussed in context of existing knowledge and generalisability to other settings |
|  | Overall quality assessment | High |  |
| Charyeva 2019 | 1. Was there a clear statement of the aims of the research? | Yes | Goal of understanding which aspects of a social support programme worked to improve treatment adherence and important because Ukraine has a high burden of MDR-TB |
|  | 2. Is a qualitative methodology appropriate? | Yes | Research seeks to explore patients' subjective experiences of the social support programme |
|  | 3. Was the research design appropriate to address the aims of the research? | Yes | Explanation as to why semi-structured interviews were used for each participant sub-group |
|  | 4. Was the recruitment strategy appropriate to the aims of the research? | Can't tell | Explanation of participant selection, through convenience sampling of nurses who then nominated some of their patients for interview. Unclear if participants selected were the most appropriate |
|  | 5. Was the data collected in a way that addressed the research issue? | Yes | Methods of data collection are explicit |
|  | 6. Has the relationship between researcher and participants been adequately considered? | No | No statement of reflexivity |
|  | 7. Have ethical issues been taken into consideration? | Yes | Explanation of how study details wre made explicit to participant and how consent was obtained. Approval sought from ethics committee |
|  | 8. Was the data analysis sufficiently rigorous? | Yes | Process of thematic analysis described, with use of two researchers. But no mention of how researchers' views impact analysis |
|  | 9. Is there a clear statement of findings? | No | Main themes are unclear and no synthesis of findings into a framework in relation to research aim i.e. which components of the intervention worked. Some triangulation between nurses' and patients' perspectives. |
|  | 10. How valuable is the research? | Can't tell | Discussion of how study informs current practice in Ukraine, but no discussion of translation in other settings |
|  | Overall quality assessment | Moderate |  |
| Davytan 2015 | 1. Was there a clear statement of the aims of the research? | Yes | Research aim stated |
|  | 2. Is a qualitative methodology appropriate? | Yes | Research seeks to identify knowledge and perceptions of TB social support programme |
|  | 3. Was the research design appropriate to address the aims of the research? | Yes | Research design justified and linked to aims |
|  | 4. Was the recruitment strategy appropriate to the aims of the research? | No | Random recruitment from TB patients and physicians who had been registered in 2013 and completed treatment; selection bias to patients who successfully completed treatment; no discussion around this strategy |
|  | 5. Was the data collected in a way that addressed the research issue? | Can’t tell | Details of in-depth interviews provided; unclear setting and form of interview and no discussion of data saturation |
|  | 6. Has the relationship between researcher and participants been adequately considered? | No | No statement |
|  | 7. Have ethical issues been taken into consideration? | Yes | Statement of participant consent and ethical approval obtained from ethics committee |
|  | 8. Was the data analysis sufficiently rigorous? | Can’t tell | Analysis process not clearly described; no participant quotes presented to support the findings; no mention of researcher reflexivity |
|  | 9. Is there a clear statement of findings? | No | Themes not explicit; no discussion of validity of findings |
|  | 10. How valuable is the research? | Yes | Qualitative findings used to contextualise the quantitative data; research used to inform Armenian TB control programme; some discussion on generalisability to other populations |
|  | Overall quality assessment | Low |  |
| George 2020 | 1. Was there a clear statement of the aims of the research? | Yes | Research aim stated |
|  | 2. Is a qualitative methodology appropriate? | Yes | Study aims to illuminate the enablers and challengers faced during provision of TB support services |
|  | 3. Was the research design appropriate to address the aims of the research? | Yes | Description and justification provided of use of in-depth interviews and FGDs |
|  | 4. Was the recruitment strategy appropriate to the aims of the research? | Can’t tell | Recruitment strategy not adequately described or justified |
|  | 5. Was the data collected in a way that addressed the research issue? | Yes | Method of data collection clearly described |
|  | 6. Has the relationship between researcher and participants been adequately considered? | No | No statement |
|  | 7. Have ethical issues been taken into consideration? | Yes | Ethical clearance obtained; no further discussion within paper |
|  | 8. Was the data analysis sufficiently rigorous? | Can’t tell | Thematic analysis described, but emerging themes not clearly stated. Unclear if |
|  | 9. Is there a clear statement of findings? | Yes | Reasonable summary of findings from complex interventions across 14 districts |
|  | 10. How valuable is the research? | Yes | Discussion of findings in context of existing knowledge and programs, with a brief discussion on generalizability |
|  | Overall quality assessment | Moderate |  |
| Horter 2020 | 1. Was there a clear statement of the aims of the research? | Yes | Research aim clearly stated |
|  | 2. Is a qualitative methodology appropriate? | Yes | Study aims to examine perceptions and experience relating to person-centered care |
|  | 3. Was the research design appropriate to address the aims of the research? | Yes | Justification of research design given |
|  | 4. Was the recruitment strategy appropriate to the aims of the research? | Yes | Purposive sampling until evidence of data saturation |
|  | 5. Was the data collected in a way that addressed the research issue? | Yes | In-depth interviews conducted with people with TB and healthcare workers |
|  | 6. Has the relationship between researcher and participants been adequately considered? | Yes | Discussion of role of interview in influencing participants’ views |
|  | 7. Have ethical issues been taken into consideration? | Yes | Ethical clearance obtained |
|  | 8. Was the data analysis sufficiently rigorous? | Yes | In-depth discussion of analysis process, derivation of themes and discrepancies |
|  | 9. Is there a clear statement of findings? | Yes | Findings are explicit |
|  | 10. How valuable is the research? | Yes | Findings discussed in context of existing knowledge and generalizability and recommendations for further research made |
|  | Overall quality assessment | High |  |
| Orlandi 2018 | 1. Was there a clear statement of the aims of the research? | Yes | Aim clearly stated |
|  | 2. Is a qualitative methodology appropriate? | Yes | Study aims to explore health workers' perspectives of the influence of social incentives on treatment adherence |
|  | 3. Was the research design appropriate to address the aims of the research? | Yes | Justification of the use of open-ended, guided interview questions |
|  | 4. Was the recruitment strategy appropriate to the aims of the research? | Can't tell | Unclear how health workers were selected and if they are representative |
|  | 5. Was the data collected in a way that addressed the research issue? | Yes | Data saturation discussed and transparency of interview questions |
|  | 6. Has the relationship between researcher and participants been adequately considered? | No | No statement |
|  | 7. Have ethical issues been taken into consideration? | Can't tell | No explicit statement of consent given, but study approved by ethics committee |
|  | 8. Was the data analysis sufficiently rigorous? | Can't tell | No statement of second researcher and no triangulation of data as only interviewing health workers, but large sample used. Not made explicit how four main themes were generated from the data |
|  | 9. Is there a clear statement of findings? | Yes | Four main themes described with adequate evidence presented |
|  | 10. How valuable is the research? | Yes | Research findings positioned within wider literature on incentives for treatment adherence in |
|  | Overall quality assessment | Moderate |  |
| Snyman 2018 | 1. Was there a clear statement of the aims of the research? | Yes | Aim clearly explained |
|  | 2. Is a qualitative methodology appropriate? | Yes | Study aims to explore experiences and perceptions of patients, supporters, health workers and program managers of the intervention |
|  | 3. Was the research design appropriate to address the aims of the research? | Can’t tell | Some discussion of why qualitative methods used |
|  | 4. Was the recruitment strategy appropriate to the aims of the research? | Yes | Purposive sampling of participants |
|  | 5. Was the data collected in a way that addressed the research issue? | Yes | Methods of data collection and forms of data are made explicit. Statement of iterative process |
|  | 6. Has the relationship between researcher and participants been adequately considered? | No | No statement |
|  | 7. Have ethical issues been taken into consideration? | Yes | Statement of consent and approval obtained from ethics committee |
|  | 8. Was the data analysis sufficiently rigorous? | Yes | Process of thematic analysis used, with two researchers involved in coding. Themes and sub-themes presented with some contradictory findings discussed |
|  | 9. Is there a clear statement of findings? | Yes | Findings are explicit and adequate evidence presented in line with study aim |
|  | 10. How valuable is the research? | Can't tell | Discussion of how research adds to existing knowledge about TB treatment interventions and makes recommendations, but limited by generalizability to other settings |
|  | Overall quality assessment | Moderate |  |
| Ukwaja 2017b | 1. Was there a clear statement of the aims of the research? | Yes | Aim clearly explained |
|  | 2. Is a qualitative methodology appropriate? | Yes | Study aims to explore patients' and health workers' subjective experiences of the intervention |
|  | 3. Was the research design appropriate to address the aims of the research? | Yes | Justification of qualitative methods used |
|  | 4. Was the recruitment strategy appropriate to the aims of the research? | Yes | Purposive sampling strategy clearly explained |
|  | 5. Was the data collected in a way that addressed the research issue? | Yes | Use and process of in-depth interviews, FGDs and semi-structured interviews justified and explained. Discussion of data saturation |
|  | 6. Has the relationship between researcher and participants been adequately considered? | Yes | Statement of researchers' role in data analysis |
|  | 7. Have ethical issues been taken into consideration? | Yes | Statement of seeking informed consent and ethical approval from ethics committee |
|  | 8. Was the data analysis sufficiently rigorous? | Yes | Process of thematic analysis clearly described and explanation of how themes were agreed upon between two researchers |
|  | 9. Is there a clear statement of findings? | Yes | Adequate discussion of evidence for findings and credibility with respect to research aim |
|  | 10. How valuable is the research? | Can't tell | Recommendations made for current practice in resource-poor settings, but limited discussion of similar studies |
|  | Overall quality assessment | High |  |
| Walker 2018 | 1. Was there a clear statement of the aims of the research? | Yes | Aim to evaluate the feasibility and acceptability of a psychosocial intervention within routine MDR-TB treatment services in Nepal. Important because gap in psychosocial support for patients with MDR-TB identified in Nepal national treatment programme |
|  | 2. Is a qualitative methodology appropriate? | Yes | In line with aims of evaluating acceptability and feasibility of a pilot intervention- able to explore the experiences and subjective views of participants |
|  | 3. Was the research design appropriate to address the aims of the research? | Can't tell | No justification of the study design |
|  | 4. Was the recruitment strategy appropriate to the aims of the research? | No | Purposive sampling based on motivation of healthcare workers to deliver intervention is not suitable for feasibility study. Explanation of eligibility criteria not given |
|  | 5. Was the data collected in a way that addressed the research issue? | Yes | Transparent explanation of data collection with semi-structured interview templates provided and discussion of data saturation |
|  | 6. Has the relationship between researcher and participants been adequately considered? | No | No critical evaluation of the role of the researcher in study design or data collection |
|  | 7. Have ethical issues been taken into consideration? | Yes | Written or verbal consent obtained from all research participants and approval sought from ethics committee |
|  | 8. Was the data analysis sufficiently rigorous? | Can't tell | Some discussion on method of thematic analysis and use of three researchers for coding. Unclear how the framework arose from the identified themes. No discussion of how researcher has influenced analysis and selection of data |
|  | 9. Is there a clear statement of findings? | Yes | Findings are adequately discussed. Limited data presented against the researcher's arguments |
|  | 10. How valuable is the research? | Yes | In-depth discussion on how findings relate to current practice in Nepal, but little discussion on relation to other studies on psychosocial interventions (but acknowledged that there are few studies on this) |
|  | Overall quality assessment | Moderate |  |
| Wingfield 2015 | 1. Was there a clear statement of the aims of the research? | Yes | States aim of outlining the operational logisitcs of implementing a TB-specific socioeconomic intervention in a low-resource settings |
|  | 2. Is a qualitative methodology appropriate? | Yes | In order to understand participants' perspectives of the intervention |
|  | 3. Was the research design appropriate to address the aims of the research? | Can't tell | No discussion on how they decided which methods to use |
|  | 4. Was the recruitment strategy appropriate to the aims of the research? | Can't tell | No statement of how or how many participants were selected |
|  | 5. Was the data collected in a way that addressed the research issue? | Can't tell | No discussion on methods used to collect data |
|  | 6. Has the relationship between researcher and participants been adequately considered? | No | No statement of reflexivity |
|  | 7. Have ethical issues been taken into consideration? | Yes | Ethical approval obtained from ethics committee and written consent obtained from participants |
|  | 8. Was the data analysis sufficiently rigorous? | Can't tell | No statement on method of data analysis or how many researchers involved |
|  | 9. Is there a clear statement of findings? | No | Findings ae presented clearly, but no discussion of evidence against researcher's arguments, limiting credibility of findings |
|  | 10. How valuable is the research? | Can't tell | Good consideration of findings in relation to current practice, but limited discussion of how findings are translatable to other settings of populations. |
|  | Overall quality assessment | Low |  |
| Yin 2018 | 1. Was there a clear statement of the aims of the research? | Yes | Research aim stated |
|  | 2. Is a qualitative methodology appropriate? | Yes | Study aimed to evaluate barriers to the implementation of the social support intervention |
|  | 3. Was the research design appropriate to address the aims of the research? | No | Data collected retrospectively, between 2-5 years post-TB treatment and limited justification of this approach |
|  | 4. Was the recruitment strategy appropriate to the aims of the research? | Can't tell | Unclear how participants were recruited from study population and whether they are representative |
|  | 5. Was the data collected in a way that addressed the research issue? | No | Only barriers to social support intervention discussed |
|  | 6. Has the relationship between researcher and participants been adequately considered? | No | No statement |
|  | 7. Have ethical issues been taken into consideration? | Yes | Statement of participant consent and ethical approval obtained from ethics committee |
|  | 8. Was the data analysis sufficiently rigorous? | Can't tell | Framework approach described but no evidence of use of framework in results. No mention of data saturation and unclear if data are sufficient to support findings |
|  | 9. Is there a clear statement of findings? | No | No clear organisation of findings and no framework as outlined in methods section. Discussion of findings do not align with results. |
|  | 10. How valuable is the research? | No | Data gives some insight into why the intervention was not successful, but does not help to make recommendations as unclear what worked |
|  | Overall quality assessment | Low |  |
