# Supplementary material for: Psychosocial support interventions to improve treatment outcomes for people living with tuberculosis: a mixed methods systematic review and meta-analysis
Source: eClinicalMedicine. 2023 Jun 27;61:102057. doi: 10.1016/j.eclinm.2023.102057 (PMC10338299; doi:10.1016/j.eclinm.2023.102057)
Supplement: Supplementary Figs. S1–S4 [file mmc3.docx]

**Contents**

[Supplementary Figure 1: Forest plots for comparison of financial support versus standard care 2](#_Toc137995492)

[Supplementary Figure 2: Forest plots for comparison of psychological-based support (health education) vs standard care 2](#_Toc137995493)

[Supplementary Figure 3: Forest plots for comparison of psychological-based support (counselling and health education) vs standard care 2](#_Toc137995494)

[Supplementary Figure 4: Forest plots for comparison of psychosocial interventions vs standard care 5](#_Toc137995495)

### Supplementary Figure 1: Forest plots for comparison of financial support versus standard care

1.1 Treatment success

### Supplementary Figure 2: Forest plots for comparison of psychological-based support (health education) vs standard care

2.2 Treatment success

### Supplementary Figure 3: Forest plots for comparison of psychological-based support (counselling and health education) vs standard care

3.1 Treatment success

3.2 Treatment failure

3.3 Death

3.4 LTFU

### Supplementary Figure 4: Forest plots for comparison of psychosocial interventions vs standard care

4.1 Treatment success

4.2 Treatment failure

4.3 Death

4.4  LTFU
