# Supplementary material for: Psychosocial support interventions to improve treatment outcomes for people living with tuberculosis: a mixed methods systematic review and meta-analysis
Source: eClinicalMedicine. 2023 Jun 27;61:102057. doi: 10.1016/j.eclinm.2023.102057 (PMC10338299; doi:10.1016/j.eclinm.2023.102057)
Supplement: Supplementary Tables S7–S11 [file mmc4.docx]

**Contents**

[Supplementary Table 7: GRADE table for comparison of financial support vs standard care for TB outcomes 2](#_Toc137995520)

[Supplementary Table 8: GRADE tables for comparison of health education vs standard care for TB outcomes 2](#_Toc137995521)

[Supplementary Table 9: GRADE tables for comparison of counselling and health education vs standard care for TB outcomes 2](#_Toc137995522)

[Supplementary Table 10: GRADE tables for comparison of psychosocial interventions vs standard care for TB outcomes 3](#_Toc137995523)

[Supplementary Table 11: GRADE assessment of qualitative themes 4](#_Toc137995524)

### Supplementary Table 7: GRADE table for comparison of financial support vs standard care for TB outcomes

| **Quality assessment** | | | | | | | **No of patients** | | **Effect** | | **Quality** |
| --- | --- | --- | --- | --- | --- | --- | --- | --- | --- | --- | --- |
| **No of studies** | **Design** | **Risk of bias** | **Inconsistency** | **Indirectness** | **Imprecision** | **Other considerations** | **Financial support** | **Standard care** | **Relative** **(95% CI)** | **Absolute** |  |
| **Treatment success** | | | | | | | | | | | |
| 4 (Durovni 2018; Klein 2019; Ukwaja 2017; Yin 2018) | NRSI (cohort studies and before and after studies) | very serious^1^ | no serious inconsistency | no serious indirectness | no serious imprecision | none | 584/708  (82.5%) | 879/1225  (71.8%) | OR 2.11 (1.45 to 3.06) | 125 more per 1000 (from 69 more to 168 more) | LOW |

^1^ Very serious risk of bias in the evidence contributing to the outcomes as per ROBINS-I

### Supplementary Table 8: GRADE tables for comparison of psychological-based support (health education) vs standard care for TB outcomes

| **Quality assessment** | | | | | | | **No of patients** | | **Effect** | | **Quality** |
| --- | --- | --- | --- | --- | --- | --- | --- | --- | --- | --- | --- |
| **No of studies** | **Design** | **Risk of bias** | **Inconsistency** | **Indirectness** | **Imprecision** | **Other considerations** | **Education** | **Standard care** | **Relative (95% CI)** | **Absolute** |  |
| **Treatment success** | | | | | | | | | | | |
| 2 (Kaplan 2016; Yin 2018) | NRSI (cohort studies and before and after studies) | very serious^1^ | no serious inconsistency | no serious indirectness | no serious imprecision | none | 9412/11488  (81.9%) | 9883/11941  (82.8%) | OR 0.96 (0.89 to 1.02) | 6 fewer per 1000 (from 17 fewer to 3 more) | LOW |

^1^ Very serious risk of bias in the evidence contributing to the outcomes as per ROBINS-I

### Supplementary Table 9: GRADE tables for comparison of counselling and health education vs standard care for TB outcomes

| **Quality assessment** | | | | | | | **No of patients** | | **Effect** | | **Quality** |
| --- | --- | --- | --- | --- | --- | --- | --- | --- | --- | --- | --- |
| **No of studies** | **Design** | **Risk of bias** | **Inconsistency** | **Indirectness** | **Imprecision** | **Other considerations** | **Counselling and education** | **Standard care** | **Relative (95% CI)** | **Absolute** |  |
| **Treatment success** | | | | | | | | | | | |
| 2 (Khachadourian 2020; Muller 2019) | randomised trials | serious^1^ | no serious inconsistency | no serious indirectness | serious^2^ | none | 229/267  (85.8%) | 236/287  (82.2%) | OR 1.28 (0.64 to 2.54) | 33 more per 1000 (from 75 fewer to 99 more) | LOW |
| **Treatment failure** | | | | | | | | | | | |
| 2 (Khachadourian 2020; Muller 2019) | randomised trials | serious^1^ | no serious inconsistency | no serious indirectness | serious^2^ | none | 19/159  (11.9%) | 19/180  (10.6%) | OR 1.17 (0.5 to 2.75) | 16 more per 1000 (from 50 fewer to 139 more) | LOW |
| **Death** | | | | | | | | | | | |
| 2 (Khachadourian 2020; Muller 2019) | randomised trials | serious^1^ | no serious inconsistency | no serious indirectness | serious^2^ | none | 19/267  (7.1%) | 21/289  (7.3%) | OR 1.00 (0.51 to 1.98) | 0 fewer per 1000 (from 34 fewer to 62 more) | LOW |
| **LTFU** | | | | | | | | | | | |
| 2 (Khachadourian 2020; Muller 2019) | randomised trials | serious^1^ | very serious^3^ | serious^4^ | serious^2^ | none | 19/427  (4.4%) | 24/400  (6%) | OR 0.63 (0.06 to 6.59) | 21 fewer per 1000 (from 56 fewer to 236 more) | VERY LOW |

^1^ Serious risk of bias in the evidence contributing to the outcomes as per ROB v2
^2^ Serious imprecision as 95% CIs include line of no effect and 'appreciable benefit' or 'appreciable harm'
^3^ Very serious inconsistency due to evidence of considerable heterogeneity (I^2^ = 89%)
^4^ Outcome is indirect as one study reported a non-standard measure of LTFU, including people who were non-adherent according to a self-reported measure, people who missed 10 or more consecutive doses of medication and people who interrupted treatment for 2 or more consecutive months

### Supplementary Table 10: GRADE tables for comparison of psychosocial interventions vs standard care for TB outcomes

| **Quality assessment** | | | | | | | **No of patients** | | **Effect** | | | **Quality** |
| --- | --- | --- | --- | --- | --- | --- | --- | --- | --- | --- | --- | --- |
| **No of studies** | **Design** | **Risk of bias** | **Inconsistency** | **Indirectness** | **Imprecision** | **Other considerations** | **Psychosocial interventions (combined material and psychological)** | **Standard care** | **Relative (95% CI)** | | **Absolute** |  |
| **Treatment success (overall estimate)** | | | | | | | | | | | | |
| 4 (Bhatt 2019; Skiles 2018; Taneja 2017; Wingfield 2017) | randomised trials, quasi-randomised trials and NRSIs (cohort studies and before and after studies) | very serious^1^ | no serious inconsistency | no serious indirectness | no serious imprecision | none | 497/624 (79.6%) | 338/559  (60.5%) | OR 2.46 (1.89 to 3.22) | | 185 more per 1,000  (from 138 more to 227 more) | LOW |
| **Treatment success (RCT and quasi- randomised trial subgroup)** | | | | | | | | | | | | |
| 2 (Taneja 2017; Wingfield 2017) | randomised trials, quasi-randomised trials | serious^2^ | no serious inconsistency | no serious indirectness | serious^3^ | none | 107/167 (64.1%) | 92/185 (49.7%) | OR 1.80 (1.17 to 2.750 | 143 more per 1,000 (from 39 more to 234 more) | | LOW |
| **Treatment success (NRSI subgroup)** | | | | | | | | | | | | |
| 2 Bhatt 2019; Skiles 2018) | NRSI | very serious^1^ | no serious inconsistency | no serious indirectness | no serious imprecision | none | 497/624 (79.6%) | 338/559  (60.5%) | OR 3.01 (2.14 to 4.25) | (195 more per 1,000 from 147 more to 233 more) | | LOW |
| **Treatment failure (overall estimate)** | | | | | | | | | | | | |
| 3 (Bhatt 2019; Skiles 2018; Wingfield 2017) | randomised trials and NRSI (cohort studies and before and after studies) | very serious^1^ | serious^4^ | no serious indirectness | serious^3^ | none | 41/592 (6.9%) | 37/521  (7.1%) | OR 1.20 (0.31 to 4.70) | | 19 fewer per 1000 (from 48 fewer to 193 more) | VERY LOW |
| **Treatment failure (RCT subgroup)** GRADE not applicable as single-study estimate. Refer to risk of bias assessment. | | | | | | | | | | | | |
| **Treatment failure (NRSI subgroup)** | | | | | | | | | | | | |
| 2 (Bhatt 2019; Skiles 2018) | NRSIs | very serious^1^ | very serious^5^ | no serious indirectness | very serious^6^ | none | 497/624 (79.6%) | 338/559  (60.5%) | OR 1.51 (0.28 to 8.07) | | 42 more per 1,000 (from 67 fewer to 366 more) | VERY LOW |
| **Death (overall estimate)** | | | | | | | | | | | | |
| 3 (Bhatt 2019; Skiles 2018; Wingfield 2017) | randomised trials and NRSI | very serious^1^ | no serious inconsistency | no serious indirectness | serious^3^ | none | 21/585 (3.5%) | 47/522  (9.0%) | OR 0.43 (0.19 to 0.95) | | 49 fewer per 1000 (from 72 fewer to 4 fewer) | VERY LOW |
| **Death (RCT subgroup)** GRADE not applicable as single-study estimate. Refer to risk of bias assessment. | | | | | | | | | | | | |
| **Death (NRSI subgroup)** | | | | | | | | | | | | |
| 2 (Bhatt 2019; Skiles 2018) | randomised trials, quasi-randomised trials and NRSIs (cohort studies and before and after studies) | very serious^1^ | no serious inconsistency | no serious indirectness | no serious imprecision | none | 497/624 (79.6%) | 338/559  (60.5%) | OR 0.31 (0.16 to 0.59) | | 73 fewer per 1,000  (from 91 fewer to 42 fewer) | LOW |
| **LTFU (overall estimate)** | | | | | | | | | | | | |
| 4 (Bhatt 2019; Skiles 2018; Taneja 2017; Wingfield 2017) | randomised trials and NRSI | very serious^1^ | Serious inconsistency^4^ | no serious indirectness | serious^3^ | none | 34/615 (5.5%) | 83/556  (1.5%) | OR 0.30 (0.10 to 0.92) | | 99 fewer per 1000 (from 132 fewer to 10 fewer) | VERY LOW |
| **LTFU (RCT subgroup)** | | | | | | | | | | | | |
| 4 (Taneja 2017; Wingfield 2017) | randomised trials, quasi-randomised trials and NRSIs (cohort studies and before and after studies) | serious^2^ | no serious inconsistency | no serious indirectness | very serious^6^ | none | 26/ 170  (15.3%) | 35/ 182 (19.2%) | OR 0.76 (0.44 to 1.34) | | 39 fewer per 1,000  (from 97 fewer to 50 more) | VERY LOW |
| **LTFU (NRSI subgroup)** | | | | | | | | | | | | |
| 4 (Bhatt 2019; Skiles 2018) | randomised trials, quasi-randomised trials and NRSIs (cohort studies and before and after studies) | very serious^1^ | no serious inconsistency | no serious indirectness | no serious imprecision | none | 8/445  (1.8%) | 48/375  (12.8%) | OR 0.13 (0.06 to 0.28) | | 110 fewer per 1,000  (from 120 fewer to 89 fewer) | LOW |

^1^ Very serious risk of bias in the evidence contributing to the outcomes as per ROBINS-I (serious risk of bias as per RoB v2)

2 Serios risk of bias in the evidence contributing to the outcomes as per RoB v2

3 Serious imprecision as 95% CIs include line of no effect and 'appreciable benefit' or 'appreciable harm'
^4^ Serious inconsistency due to evidence of considerable heterogeneity, partially explained by sub-group analysis by study design

^5^ Very serious inconsistency due to evidence of considerable heterogeneity

^6^ Very serious imprecision as 95% CIs include line of no effect and 'appreciable benefit' and 'appreciable harm'

### Supplementary Table 11: GRADE assessment of qualitative themes

| **Theme** | **Contributing Studies** | **Quality assessment** | | **Quality** |
| --- | --- | --- | --- | --- |
| Improved access to care | Burtscher 2020, Charyeva 2019, Orlandi, Snyman 2018, Ukwaja 2017b | Methodological limitations | Moderate concerns as per CASP quality appraisal | **Moderate** |
|  |  | Coherence | No or very minor concerns |  |
|  |  | Adequacy | Minor concerns: the theme was based on 5 studies, 3 of which provided rich data |  |
|  |  | Relevance | No or very minor concerns |  |
| Knowledge fosters autonomy | Charyeva 2019, Davytan 2015, Horter 2020, Snyman 2018, Walker 2018 | Methodological limitations | Moderate concerns as per CASP quality appraisal | **Moderate** |
|  |  | Coherence | Minor concerns: some disagreement within 1 study whether health information is motivating due to autonomy or due to fear |  |
|  |  | Adequacy | Minor concerns: the theme was based on 5 studies, 3 of which provided rich data |  |
|  |  | Relevance | No or very minor concerns |  |
| Improved mental health | Walker 2018 | Methodological limitations | Moderate concerns | **Low** |
|  |  | Coherence | No or very minor concerns |  |
|  |  | Adequacy | Serious concerns: the theme was based on findings from 1 study which provided very thin data |  |
|  |  | Relevance | No or very minor concerns |  |
| Convenient care and flexible delivery | Burtscher 2020 , Charyeva 2019 | Methodological limitations | Minor concerns | **High** |
|  |  | Coherence | No or very minor concerns |  |
|  |  | Adequacy | Minor concerns: the theme was based on findings from 2 studies which both provided rich data |  |
|  |  | Relevance | No or very minor concerns |  |
| Connectedness and optimism | Burtscher 2020, Charyeva 2019, George 2020, Orlandi 2019, Snyman 2018, Walker 2018, Wingfield 2015 | Methodological limitations | Moderate concerns as per CASP quality appraisal | **Moderate** |
|  |  | Coherence | No or very minor concerns |  |
|  |  | Adequacy | Minor concerns: the theme was based on findings from 7 studies, 2 of which provided rich data |  |
|  |  | Relevance | No or very minor concerns |  |
| Addressing material needs | Orlandi 2019,  Ukwaja 2017b | Methodological limitations | Moderate concerns as per CASP quality appraisal | **Low** |
|  |  | Coherence | No or very minor concerns |  |
|  |  | Adequacy | Moderate concerns: the theme was based on findings from 2 studies, neither of which provided rich data |  |
|  |  | Relevance | No or very minor concerns |  |
| Addressing TB-related stigma | Ukwaja 2017b, Burtscher 2020 | Methodological limitations | Minor concerns as per CASP quality appraisal | **Moderate** |
|  |  | Coherence | No or very minor concerns |  |
|  |  | Adequacy | Moderate concerns: the theme was based on findings from 2 studies, none of which provided rich data |  |
|  |  | Relevance | No or very minor concerns |  |
| Economic empowerment | Orlandi 2019, Ukwaja 2017b, Wingfield 2015 | Methodological limitations | Moderate concerns as per CASP quality appraisal | **Low** |
|  |  | Coherence | No or very minor concerns |  |
|  |  | Adequacy | Moderate concerns: the theme was based on findings from 3 studies, 1 of which provided rich data |  |
|  |  | Relevance | No or very minor concerns |  |
| Patient-centered care | Burtscher 2020, Charyeva 2019, Orlandi 2019, Snyman 2018, Ukwaja 2017b, Walker 2019 | Methodological limitations | Moderate concerns as per CASP quality appraisal | **Moderate** |
|  |  | Coherence | No or very minor concerns |  |
|  |  | Adequacy | Minor concerns: the theme was based on findings from 5 studies, 3 of which provided rich data |  |
|  |  | Relevance | No or very minor concerns |  |
| Multi-dimensional support and integrated care | Davytan 2015, Wingfield 2015 | Methodological limitations | Moderate concerns as per CASP quality appraisal | **Low** |
|  |  | Coherence | No or very minor concerns |  |
|  |  | Adequacy | Moderate concerns: the theme was based on findings from 2 studies, neither of which provided rich data |  |
|  |  | Relevance | No or very minor concerns |  |
| Inadequate of inappropriate support | Davytan 2015, George 2020, Orlandi 2019, Wingfield 2015, Yin 2018 | Methodological limitations | Moderate concerns as per CASP quality appraisal | **Moderate** |
|  |  | Coherence | No or very minor concerns |  |
|  |  | Adequacy | Minor concerns: the theme was based on findings from 5 studies, 3 of which provided rich data |  |
|  |  | Relevance | No or very minor concerns |  |
| Implementation delays | George 2020, Wingfield 2015 | Methodological limitations | Moderate concerns as per CASP quality appraisal | **Low** |
|  |  | Coherence | No or very minor concerns |  |
|  |  | Adequacy | Moderate concerns: the theme was based on findings from 2 studies, neither of which provided rich data |  |
|  |  | Relevance | No or very minor concerns |  |
| Resource constraints | George 2020, Walker 2019 | Methodological limitations | Minor concerns as per CASP quality appraisal | **Low** |
|  |  | Coherence | Moderate concerns as per CASP quality appraisal |  |
|  |  | Adequacy | Serious concerns: the theme was based on findings from 2 studies which provided very thin data |  |
|  |  | Relevance | No or very minor concerns |  |
